# Cancer Vaccines: Mechanisms, Clinical Applications, Challenges, and Future Directions in Precision Medicine

**DOI:** 10.2174/0115680096381121250628142857

**Published:** 2025-07-11

**Authors:** Alaa A. A. Aljabali, Omar Gammoh, Mohammad Obeid, Esam Qnais, Abdelrahim Alqudah, Mohamed El-Tanani, Taher Hatahet

**Affiliations:** 1 Faculty of Pharmacy, Department of Pharmaceutics & Pharmaceutical Technology, Yarmouk University, Irbid, 21163, Jordan;; 2 Department of Clinical Pharmacy and Pharmacy Practice, Faculty of Pharmacy, Yarmouk University, PO BOX 566, Irbid, 21163, Jordan;; 3 Department of Biology and Biotechnology, Faculty of Science, The Hashemite University, Zarqa, Jordan;; 4 Department of Clinical Pharmacy and Pharmacy Practice, Faculty of Pharmaceutical Sciences, The Hashemite University, Zarqa, 13133, Jordan;; 5 College of Pharmacy, Ras Al Khaimah Medical and Health Sciences University, Ras Al Khaimah, UAE;; 6 School of Pharmacy, Queens University Belfast, 97 Lisburn Road, Belfast, BT 9 7BL, UK

**Keywords:** Personalized immunotherapy, RNA vaccines, tumor-specific antigens, cancer vaccines, immune checkpoint inhibitors

## Abstract

Cancer poses a major health burden worldwide, necessitating the development of novel therapeutic approaches. Personalized cancer vaccines represent a promising form of immunotherapy that enhances the ability of the immune system to recognize and destroy tumor cells through tumor-associated and cancer-specific antigens. This review categorizes cancer vaccines into preventive, therapeutic, and personalized vaccines, discussing their mechanisms, clinical applications, and current FDA-approved examples, such as Sipuleucel-T and HPV vaccines. We highlight the recent advances in RNA-based vaccines, viral vectors, and nanotechnology, along with the synergistic role of cancer vaccines and immune checkpoint inhibitors in improving therapeutic efficacy. Overcoming ethical, regulatory, and technological barriers through global collaboration is essential for maximizing vaccine efficacy and enhancing patient outcomes. This review highlights the pivotal role of personalized vaccines in advancing precision medicine and reshaping cancer treatment paradigms.

## 1 INTRODUCTION

Despite its success, cancer remains one of the most life-threatening diseases globally, in addition to therapies that have less success and some adverse effects in chemotherapy and radiotherapy due to tumor resistance. Cancer has a significant global impact, emerging as the primary cause of mortality worldwide, with approximately 10 million deaths attributed to the disease by 2020 (Fig. **1**) [1]. Among other approaches that have been sought to evoke an immune system to kill tumor cells are immunotherapeutic methods, especially tumor vaccines [2-4]. Cancer vaccines utilize tumor-specific and tumor-associated antigens to increase the chances of immune recognition compared with other conventional therapeutic modalities [5, 6].

Recent breakthroughs in RNA vaccines, viral vectors, and nanotechnology have accelerated the development of personalized cancer vaccines tailored according to an individual’s treatment profile. Tumor heterogeneity, immune evasion, and regulatory bottlenecks are crucial impediments in this treatment modality [7]. Vaccines are classified into preventive, therapeutic, and personalized types, highlighting their mechanisms, clinical applications, and prospects. Furthermore, it explores emerging technologies and challenges, underlining the role of personalized vaccines in driving precision medicine.

This review was conducted by systematically searching PubMed and Scopus for literature on personalized immunotherapy, RNA vaccines, tumor-specific antigens, cancer vaccines, and immune checkpoint inhibitors. Articles were selected based on predefined inclusion criteria, prioritizing relevance, recent advancements, and citations. Screening and data extraction were performed to synthesize key findings and identify emerging trends in cancer immunotherapy.

Global estimates from the World Cancer Research Fund International suggest that approximately 18,095,716 individuals are diagnosed with cancer annually. In the UK, approximately 19,000 cancer-related incidents have been reported, with an incidence rate of 433.9 and a mortality rate of 124.9 per 100,000 individuals, with a higher mortality rate in males compared to females [8]. Denmark has the highest incidence of cancer, with 334.9 cases per 100,000 individuals, followed by other nations such as Ireland, Belgium, Hungary, France, the Netherlands, Australia, Norway, France (New Caledonia), and Slovenia, all exceeding 300 cases per 100,000 individuals [8, 9]. In the United States, the American Cancer Society estimates that there will be 2,001,140 new cancer cases and 611,720 cancer-related deaths by 2024, with a notable male predominance in cancer-related mortality [10]. In Jordan, a significant health burden is attributed to cancer, with 11,559 new cases and 6,190 deaths reported, highlighting its substantial impact on overall mortality [11]. These alarming statistics underscore the urgent need for innovative strategies in cancer therapy. One promising direction is the development of cancer vaccines that harness the immune system’s capacity to recognize and eliminate malignant cells. By targeting tumor-specific antigens, such vaccines have the potential to reduce cancer burden and enhance patient survival outcomes.

The previous year, Jordan grappled with a substantial health burden. The Jordanian nation has faced numerous non-communicable diseases, with cancer being particularly prominent, as reported by the International Agency for Research on Cancer (IARC) [12]. Approximately 11,559 new cancer cases were diagnosed, with a consequent sharp increase in deaths to 6,190. Cancer-related deaths contributed to 3,084–19,676 registered deaths up to that year, eloquently testifying to their seriousness, and their contributions generally led to mortality. Notably, the male-to-female ratio of cancer-related deaths was 1.2:1 [13].

Vaccines are a promising tool for cancer treatment. They engage the immune system to elicit responses against tumor antigens, thereby preventing cancer development [14]. Similar to classical vaccines against infectious diseases, there are bright prospects for revolution in the field of public health by producing vaccines against cancers of viral origin or those of other known etiologies. Cancer vaccines represent a unique immunotherapy strategy [15]. They can be tailored to a person’s tumor profile by identifying neoantigens (mutated proteins unique to cancer cells that are recognizable by the immune system) that are characteristic of the cancer. This personalized approach results in a selective immune response against cancer cells with long-lasting and negligible side effects [16]. Cancer vaccines can be combined with other immunotherapies, such as immune checkpoint inhibitors, to enhance anti-tumor immune responses. These combination strategies show promise for improving the efficacy of cancer treatment and are increasingly being explored in the management of various other diseases [17]. Research on the development of cancer vaccines has focused on the use of novel adjuvants and delivery systems to create more potent and effective immune responses than their conventional counterparts [18]. To ensure an efficient and potent immune response through vaccination, current research has focused on nanoparticles, viral vectors, and RNA-based approaches, using RNA as molecules.

Clinical trials have yielded critical insights for optimizing cancer immunotherapy strategies. Cancer vaccines have played a pivotal role in advancing the field and represent a promising tool in the ongoing battle against cancer. With sustained research efforts, cross-disciplinary collaboration, and long-term commitment, the prospect of effective cancer control becomes increasingly attainable [19, 20]. Fig. (**2**) illustrates immune-related gene interactions, highlighting the complexity of tumor heterogeneity and immune evasion mechanisms.

### 1.1 The Concept of Cancer Vaccines and Their Potential in Cancer Treatment

Cancer vaccines are paving the way for promising advances, empowering the immune system to fight cancer with renewed vigor [21-23]. Unlike traditional prophylactic vaccines developed against infectious diseases, which elicit broad, non-specific immune responses, cancer vaccines are characterized by their precision (Fig. **3**). By targeting tumor-associated antigens, they induce a focused and robust immune response [24, 25]. Cancer vaccines represent a milestone in immunotherapy, harnessing the body's cellular immune system to identify and thwart cancerous cells or to impede their proliferation [26]. Their arrival signifies a new age in therapy, with increased effectiveness and reduced toxicity compared with chemotherapy and radiation therapy. Immunocancer vaccines are non-invasive and can be delivered in a clinical setting, changing cancer therapy forever [22, 27, 28].

Advancements in oncology and immunology have facilitated the development of cancer vaccines that target specific tumor-associated markers and stimulate antigen-specific immune responses [27]. Some of these vaccines include Sipuleucel-T (Provenge) for advanced prostate cancer, in which a patient's immune cells are engineered in the laboratory to recognize and target prostatic acid phosphatase (PAP), a protein expressed in prostate cancer cells [29]. Gardasil and Cervarix, specifically against cervical cancer, also confer protection against the human papillomavirus (HPV) responsible for most cases of genital warts [30]. There are different types of vaccines under development for melanoma; some are peptide-based, whereas others are dendritic cell-based and generated against different types of antigens specific to melanoma, such as MART-1 or gp100 [31]. However, responses to cancer vaccines vary across cancer types and individual patient characteristics. Key challenges include tumor heterogeneity, immune evasion mechanisms employed by cancer cells, and host-related factors that influence vaccine efficacy. Although ongoing clinical trials have yielded encouraging results, further research is necessary to optimize vaccine design, identify suitable patient populations, and overcome immune escape pathways. In conclusion, while cancer vaccines hold substantial therapeutic promise, extensive studies are required to refine this strategy and enhance its effectiveness in cancer management [28].

### 1.2 Mechanisms of Action: How Cancer Vaccines Work

The use of modal behavior to treat cancer has been emphasized in the field of cancer vaccines. Therapeutic or immuno-oncology vaccines include substances that aim to educate the immune system to identify and eliminate cancer cells [22, 24, 26-28, 32]. Comprising tumor antigens, immune adjuvants, and delivery systems, these formulations can provoke robust targeted immune responses against cancer [23, 24, 26].

These carefully designed biological agents aim to strengthen the body’s defense mechanisms against cancer cells. They utilize various forms and materials specifically tailored to achieve their therapeutic goals [33]. Cancer vaccines typically involve modified whole cancer cells that are cultivated under laboratory conditions. These cells are then weakened or rendered nonviable to prime the immune system to identify a broad spectrum of cancerous materials [34]. In contrast, tumor antigens derived from specific proteins on cancer cell surfaces adopt a more targeted approach, honing molecules related to cancer for tailored therapeutic interventions [35]. Some vaccines are based on dendritic cells, a type of immune cell crucial for supporting other immune system components. Dendritic cell vaccination has demonstrated potent efficacy and a highly specific immune response [36, 37].

Several new vaccines are currently being developed to combat the deadliest and most widespread disease globally. Administration routes vary depending on the vaccine composition and the recipient's health status, including injection, oral ingestion, and skin puncture [28, 38, 39]. Thus, a specially developed breast cancer vaccine offers a treatment that can be improved in terms of effectiveness and specificity, while being less toxic than existing options. This indicates a positive trend in disease control [28, 40]. Given the ongoing research and trials, the field of cancer vaccines is continuously advancing. The goal is to enhance their efficacy and expand their applications, which holds promise for future cancer therapies [19]. Recent advancements in vaccine platforms, including RNA-based vaccines, viral vectors, and nanotechnology, have significantly influenced the development of personalized anticancer therapies [41, 42]. These next-generation technologies improve antigen delivery, enhance immune activation, and extend the duration of immune system engagement [43]. In addition, the concurrent administration of cancer vaccines with immune checkpoint inhibitors, such as anti-PD-1 and anti-PD-L1 therapies, has demonstrated efficacy in overcoming tumor-induced immune suppression and enhancing overall therapeutic outcomes. This review presents a critical evaluation of these emerging strategies and explores their potential to redefine current approaches to anticancer treatment. [44]. Fig. (**4**) shows a linear interaction model that describes the key mediators of the immune system involved in the activity of cancer vaccines. This illustrates how cancer vaccines target specific processes, such as processing through MHC molecules and the activation of cytotoxic T cells, thereby enhancing the immune system's ability to detect and destroy tumor cells.

### 1.3 Precision and Proactivity in Cancer Treatment: Cancer Vaccines

Cancer vaccines represent a major advancement in cancer treatment due to their focused approach, potentially reducing toxicity compared to standard chemotherapy and radiation. Unlike conventional therapies, which can harm healthy tissues and cancer cells, causing side effects, cancer vaccines arm the immune system to recognize and attack cancer cells [28]. This largely reduces collateral damage caused by attacking healthy tissues, while causing fewer adverse effects. Cancer vaccines can also induce long-term immune memory and eliminate existing cancer cells. The immune system "remembers" cancer cells and can be easily called upon to defend when cells reappear, prolonging protection from recurrence [45]. Cancer vaccines represent a departure from reactive treatment approaches targeting cancer after occurrence, turning towards initiative-taking strategies to prevent cancer establishment and recurrence. This prevention provides a huge leap from traditional treatment, which mostly relates to disease management [16]. Various cancer vaccines are currently in research or clinical application, with different mechanisms of action. Prophylactic vaccines target cancer-causing viruses, such as HPV and hepatitis B virus (HBV), to prevent cancer by preventing infection. Therapeutic vaccines help the immune system recognize and destroy existing cancer cells. [28]. Examples include peptides, dendritic cells, and tumor cell vaccines. In addition to these promises, cancer vaccines face several challenges, such as tumor heterogeneity and evasion from the immune system. Currently, most research has focused on optimizing the design of vaccines and trying to find the best way to treat specific cancer types [34]. Cancer vaccination has opened a perspective on a new method for treating cancer more precisely, effectively, and with less toxicity. In particular, the evocation of long-lasting immune memory that prevents cancer recurrence is a serious breakthrough in the treatment and prophylaxis of cancer [46].

Various cancer vaccines possess distinct mechanisms and targets.

1. **Peptide-Based Vaccines**: Utilizing short protein fragments (peptides) derived from tumor-associated antigens, these vaccines provoke an immune response [47,48].
2. **Dendritic Cell-based vaccines**: using dendritic cells are powerful antigen-presenting cells that activate T cells against cancer antigens [36, 37].
3. **DNA and RNA Vaccines**: Delivering genetic material that encodes tumor antigens and instructing the immune system to identify and combat cancer cells [42,49-51].
4. **Viral Vector Vaccines**: Modified viruses function as delivery vehicles for cancer antigens, triggering a robust immune response [52-54].

Each cancer vaccine type has unique strengths and limitations, emphasizing the importance of a personalized approach based on specific cancer characteristics and patient profile, as shown in Table **1**. Although the potential of cancer vaccines remains unclear, further investigation is needed. Tumor heterogeneity, which refers to variations in antigen expression among cancer cells within a single tumor, is a significant hurdle [21]. In addition, tumors commonly exert immune evasion strategies against which the host cannot react [55]. These countermeasures can be improved by advances in precision medicine and immunology. Individualized cancer vaccines, unique to an individual's tumor profile, hold great promise [56]. Moreover, combination therapies consisting of a mixture of checkpoint inhibitors have immense potential to enhance the therapeutic efficacy [17, 20]. Combination therapies, such as pairing cancer vaccines with checkpoint inhibitors, are one way to increase the therapeutic efficacy.

These are all valid cancer vaccine approaches, but they differ in platform, antigen delivery strategy, and immunological targeting. However, for continued growth, one must consider their limitations and complexities. A vaccine against cancer may be evaded by the immune system of the cancer cell; immune-evasive cancer cells limit the efficacy of the vaccine, as shown in Fig. (**5**). Some strategies considered by researchers include combining vaccines with checkpoint inhibitors to overcome immune suppression by cancer cells. For example, in some patients with advanced melanoma, a personalized neoantigen vaccine has been combined with the checkpoint inhibitor pembrolizumab [57]. An example of this type of vaccine is the personalized neoantigen vaccine NeoVax, which was developed based on specific mutations identified in a patient's tumor to target cancerous cells [58]. While the vaccine primes the immune system to recognize cancer cells, the checkpoint inhibitor releases the brakes on inhibitory signals from cancer cells evading immune attack. Early clinical trials using this approach for several cancers, including lung cancer and melanoma, have shown good results [59].

Another area of intensive research is exploring combinations with other immunotherapies, including CAR-T cell therapy. Such multidimensional approaches should act synergistically to increase immune reactivity against cancer beyond levels achievable with monotherapy [60]. The idea of a personalized cancer vaccine is to use the NeoVax approach to identify and target mutations unique to a patient's tumor. These studies showed great promise for the use of vaccines applying this approach, even in early stage clinical trials for melanoma and glioblastoma, among others [61]. Moderna and BioNTech have positioned themselves at the cutting edge of developing mRNA vaccines to fight infectious diseases, and have used these technological breakthroughs to develop cancer immunotherapies. Personalized mRNA-based cancer vaccines combine next-generation sequencing with bioinformatics analysis to identify individual neoantigens present in cancer patients. Targeted mRNA vaccines encode individual neoantigens that direct the immune system to launch specific attacks. Notably, both Moderna’s mRNA-4157 and BioNTech’s BNT122 have moved into melanoma and non-small cell lung cancer trials with early positive indications, when delivered in combination with immune checkpoint inhibitors. Personalized cancer vaccines represent a significant breakthrough in the field of precision oncology, delivering individualized therapeutic options designed for specific tumor markers [62].

Cancer is a complex disease caused by genetic mutations leading to abnormal cell growth and uncontrolled proliferation, resulting in tumor formation. These cancer cells can metastasize through the bloodstream or lymphatic system [2-4]. Cancer treatment involves various approaches, including surgery, chemotherapy, and radiation therapy. Emerging therapies, such as immunotherapy and targeted treatment, are becoming more important, changing the way cancer is treated, compared with older methods [5, 6]. The most prevalent types of cancer are lung, breast, colon, prostate, and skin cancer, each typically exhibiting distinct incidence rates across different geographical regions [7].

Global estimates from the World Cancer Research Fund International suggest that approximately 18,095,716 individuals worldwide are diagnosed with cancer annually. The UK has witnessed approximately 19,000 cancer-related incidents of 433.9 with a mortality rate of 124.9 per 100,000 individuals. Notably, the mortality rate of cancer in the UK generally favors males over females [8]. Denmark has the highest incidence of cancer, with 334.9 cases per 100,000 individuals. Other nations surpassing 300 per 100,000 include Ireland, Belgium, Hungary, France, the Netherlands, Australia, Norway, France (New Caledonia), and Slovenia [8, 9]. The American Cancer Society estimates new cancer cases and deaths in the US annually, compiling recent data on cancer occurrence and outcomes through 2020 using incidence data from NCI, NPCR, SEER registries, and mortality data from the National Center for Health Statistics. It is estimated there will be 2,001,140 new cancer cases and 611,720 cancer deaths in the US in 2024 [10]. Additionally, it was projected that approximately 608,570 Americans would succumb to cancer in that year. While explicit data on the male-to-female ratio of cancer mortality in the U.S. are lacking, cancer-related deaths are more prevalent among males [11].

The previous year, Jordan grappled with a substantial health burden. The Jordanian nation has faced numerous non-communicable diseases, with cancer being particularly prominent, as reported by the International Agency for Research on Cancer (IARC) [12]. Approximately 11,559 new cancer cases were diagnosed, leading to a significant increase in deaths, reaching 6,190. The fact that 3,084 of the 19,676 registered deaths up to that year were due to cancer puts the situation into perspective and provides evidence of their significant contribution to total mortality. The proportion of male to female cancer-related deaths is 1.2:1 [13].

Vaccines are a promising tool for cancer treatment. Vaccines activate the immune system to target specific tumor antigens and can therefore prevent the incidence of cancer [14]. These cancer vaccines, similar to classic vaccines against infectious diseases, could affect public health by changing the landscape of vaccination against viral cancers or other known etiologies. Cancer vaccines represent a unique approach to immunotherapy. They can be tailored to an individual's specific tumor profile using neoantigens characteristic of cancer [15]. A customized approach may lead to selective immune responses against cancer cells and long-term positive effects, with minimal side effects [16].

Cancer vaccines can be combined with other immunotherapies, like checkpoint inhibitors, to enhance immune response against cancer. These combination strategies provide hope for better cancer treatment and are applied to several other diseases [17]. Novel adjuvants and delivery systems were used in the present study. Nanoparticles, viral vectors, and RNA-based approaches-all based on RNA-are being developed to provide a potent, efficient immune response to vaccines [18]. Clinical trials have refined strategies. Cancer vaccines have significantly advanced science and become a beacon of hope in the fight against cancer. With continued research, collaboration, and dedication, we can envision a future where cancer management is possible [19, 20].

### 1.4 The Concept of Cancer Vaccines and Their Potential in Cancer Treatment

Cancer vaccines are paving the way for promising advances, empowering the immune system to fight cancer with renewed vigor [21-23]. Unlike earlier prophylactic vaccines that targeted infectious diseases and elicited a generalized immune response, precision is a hallmark of cancer vaccines. By specifically targeting cancer cell antigens, they provoke a focused and potent immune response [24, 25]. Cancer vaccines represent a milestone in immunotherapy, harnessing the body's cellular immune system to identify and thwart cancerous cells or to impede their proliferation [26]. Their advent heralds a new era in treatment, offering enhanced efficacy with reduced toxicity compared to conventional methods like chemotherapy and radiation therapy. The non-invasive nature of cancer vaccines facilitates their administration in clinical settings, highlighting their potential as a novel frontier in cancer treatment [22, 27, 28].

Immunological and oncological advancements now allow for the design of cancer vaccines that target specific tumor markers. These markers are molecules that are present on the surface of cancer cells. This design primarily involves engaging the immune system to recognize and fight cancerous cells bearing these markers [27, 27]. As described earlier, Sipuleucel-T (Provenge) is an FDA-approved vaccine indicated for advanced prostate cancer, while Gardasil and Cervarix are used to prevent HPV-associated malignancies. These examples underscore the importance of antigen-specific immune activation in both cancer prevention and therapeutic intervention [29]. Gardasil and Cervarix, specifically against cervical cancer, also confer protection against the human papillomavirus (HPV) responsible for most cases of genital warts [30]. There are different types of vaccines under development for melanoma: some are peptide-based, whereas others are dendritic cell-based and generated against different types of antigens specific to melanoma, such as MART-1 or gp100 [31]. However, responses to cancer vaccines differ among different cancers and patient characteristics. Although current clinical trials are ongoing and promising results have been obtained, further research is needed to improve the design of vaccines, define optimal candidates, and approach problems such as immune escape. In conclusion, although cancer vaccines have potential, it is pertinent that many studies have been conducted to fine-tune this new strategy and increase its effectiveness in treating cancers [28].

### 1.5 Mechanisms of Action: How Cancer Vaccines Work

The use of modal behavior to treat cancer has been emphasized in the field of cancer vaccines. Therapeutic or immuno-oncology vaccines include substances that aim to educate the immune system to identify and eliminate cancer cells [22, 24, 26-28, 32]. Comprising tumor antigens, immune adjuvants, and delivery systems, these formulations can provoke robust targeted immune responses against cancer [23, 24, 26].

These biological agents aim to strengthen the body's defense mechanisms against cancer cells. They utilize various forms and materials tailored to achieve their therapeutic goals [33]. Cancer vaccines typically involve modified whole cancer cells cultivated under laboratory conditions. These cells are then weakened or rendered nonviable to prime the immune system to identify a broad spectrum of cancerous materials [34]. In contrast, tumor antigens derived from specific proteins on cancer cell surfaces adopt a more targeted approach, honing molecules related to cancer for tailored therapeutic interventions [35]. Some vaccines are based on dendritic cells, a type of immune cell crucial for supporting other immune system components. Dendritic cell vaccines represent a potent form of cancer immunotherapy that enhances antigen presentation and induces robust T cell responses. The clinical efficacy of Sipuleucel-T, the first FDA-approved dendritic cell-based vaccine, underscores the therapeutic potential of this platform in oncology. [36, 37].

Several new vaccines are being developed to combat the deadliest and most widespread disease globally. Administration routes vary depending on vaccine composition and the recipient's health status, including injection, oral ingestion, and skin puncture [28, 38, 39]. Thus, a specially developed breast cancer vaccine offers a treatment that can be improved in terms of effectiveness and specificity, while being less toxic than existing options. This indicates a positive trend in disease control [28, 40]. Given the ongoing research and trials, the field of cancer vaccines is continuously advancing. The goal is to enhance their efficacy and expand their applications, which holds promise for future cancer therapies [19]. RNA-based cancer vaccines employ synthesized messenger RNA encoding tumor-associated antigens to elicit strong and adaptable immune responses, offering high potential for personalized therapeutic applications in oncology [63, 64]. Viral vector vaccines utilize genetically engineered viruses to deliver antigens directly to antigen-presenting cells, thereby enhancing immune system activation [43]. Nanotechnology-based delivery systems, including polymer carriers and lipid nanoparticles, improve vaccine stability during antigen presentation and enhance immunogenicity while minimizing off-target toxicity. Collectively, these emerging technologies represent promising alternatives to conventional vaccine platforms and have recently advanced to clinical trials in humans. [65, 66]. Fig. (**6**) provides a comprehensive overview of the immune checkpoint pathways, highlighting the PD-1/PD-L1 interactions, downstream signaling events, and therapeutic inhibitors targeting these pathways.

The integration of immune checkpoint inhibitors into cancer vaccine regimens has emerged as a promising strategy for enhancing antitumor immune responses [67]. Checkpoint blockade agents, such as PD-1/PD-L1 and CTLA-4 inhibitors, eliminate inhibitory immune signals and enable effective T cell–mediated targeting and destruction of cancer cells following vaccine-induced activation [44, 68]. Emerging clinical evidence indicates that patients receiving combination therapy with checkpoint inhibitors and cancer vaccines exhibit improved clinicopathologic outcomes, prolonged progression-free survival, and enhanced immune memory against neoplastic cells. [17, 67, 69].

### 1.6 Precision and Proactivity in Cancer Treatment: Cancer Vaccines

Cancer vaccines represent a major advance in cancer treatment due to their focused approach, potentially reducing toxicity compared to standard chemotherapy and radiation. Unlike conventional therapies, which can damage both healthy tissues and cancer cells, causing harmful side effects, cancer vaccines arm the immune system to recognize and specifically attack cancer cells [28]. This largely reduces the collateral damage that could be caused by attacking healthy tissues, while simultaneously causing fewer adverse effects. Cancer vaccines can also induce long-term immune memory and eliminate existing cancer cells. In this case, the immune system "remembers" cancer cells and can be easily called upon to take up a defense when and if the cells reappear, thereby prolonging protection from recurrence [45]. Cancer vaccines are, therefore, a departure from the previous reactive treatment approaches, which target cancer after it has occurred, and a turn towards initiative-taking strategies that will prevent the establishment and recurrence of cancer. Such prevention provides a huge leap toward the traditional treatment currently in use, which is mostly related to disease management [16]. Various cancer vaccines are currently in research or clinical application, but each of these vaccines has a different mechanism of action. Prophylactic vaccines target cancer-causing viruses, such as HPV and hepatitis B virus (HBV), to prevent the occurrence of cancer by preventing infection. Therapeutic vaccines help the immune system recognize and destroy cancer cells that already exist [28]. Examples include peptides, dendritic cells, and tumor cell vaccines. Cancer vaccines face challenges such as tumor heterogeneity and immune system evasion. Currently, research focuses on optimizing vaccine design and finding the best treatment for specific cancer types [34]. Cancer vaccination has opened a perspective on a new method of treating cancer more precisely, effectively, and with less toxicity. In particular, the evocation of long-lasting immune memory that prevents cancer recurrence is a serious breakthrough in the treatment and prophylaxis of cancer [46]. Cancer vaccines are generally classified into four main categories: preventive, therapeutic, peptide-based, and dendritic cell-based formulations.

Each cancer vaccine type has unique strengths and limitations, emphasizing the importance of a personalized approach based on specific cancer characteristics and patient profile, as shown in Table **2**. Although the potential of cancer vaccines remains unclear, further investigation is needed. Tumor heterogeneity, which refers to variations in antigen expression among cancer cells within a single tumor, is a significant hurdle [42, 70, 71].

Moreover, tumors work based on immune evasive concepts that make their chances of survival insurmountable to the immune cell response [55]. Advances in precision medicine and immunology have provided opportunities to overcome these challenges. According to their eventual profile, custom-personalized cancer vaccines were established to be efficacious by Kiyotani *et al.* 2021 [56]. The investigations of combination therapies, for instance, those involving cancer vaccines and checkpoint inhibitors, however, hold a huge potential to improve the therapeutic efficacy [17, 20]. Combination therapies, such as the combination of cancer vaccines and checkpoint inhibitors, may be particularly useful for increasing therapeutic effectiveness.

These are good but different vaccines against cancer; however, for continued growth, one must consider their limitations and complexities. A vaccine against cancer may be evaded by the immune system of the cancer cells; immune-evasive cancer cells limit the efficacy of the vaccine, as shown by research publications. Strategies considered by researchers include combining vaccines with checkpoint inhibitors to overcome immune suppression by cancer cells. For example, in some patients with advanced melanoma, a personalized neoantigen vaccine has been combined with the checkpoint inhibitor pembrolizumab [57]. Another critical issue is tumor heterogeneity: different cancer cells in the tumor express various antigens that cannot be targeted by one vaccine. A personalized cancer vaccine based on a tumor's unique profile is currently being developed. An example is the personalized neoantigen vaccine NeoVax, developed to target cancerous cells according to specific mutations identified in a patient's tumor [58]. One of the most important strategies in this area is combining different therapies to increase efficacy. The most promising regimen combines cancer vaccines and checkpoint inhibitors to generate stronger immune responses against cancer. The vaccine primes the immune system to recognize cancer cells, while the checkpoint inhibitor releases the brakes on inhibitory signals from cancer cells that evade immune attack. Early clinical trials using this approach for several cancers, including lung cancer and melanoma, have shown good results [59]. Another area of intensive research is the exploration of combinations with other immunotherapies, including CAR-T cell therapy. Such multidimensional approaches should act synergistically to raise immune reactivity against cancer to levels at which monotherapy may not be effective [60]. The idea of a personalized cancer vaccine is to use the NeoVax approach to identify and target mutations unique to a patient's tumor. These showed great promise for the use of vaccines applying this approach, even in early-stage clinical trials for melanoma and glioblastoma, among others [61]. Companies like Moderna and BioNTech are developing personalized mRNA cancer vaccines tailored to individual tumor mutations. These vaccines provide patients' cells with genetic instructions to create tumor antigens, stimulating an immune response. This marks cancer vaccine research as a dynamic area evolving to face challenges and develop therapeutic efficacy [62]. Cancer vaccines hold high potential to revolutionize cancer care and patient outcomes through individualized treatment and combination therapies with continuous innovation.

## 2 MILESTONES IN CANCER VACCINE DEVELOPMENT

The development and clinical application of cancer vaccines and immunotherapy have a rich historical background. This dynamic, fast-evolving research topic is marked by advancements and challenges. Over the years, these vaccines have seen both success and failure [72].

Advances in knowledge and applied science have fuelled efforts to improve vaccine effectiveness and patient outcomes. Recent research on exosome-based vaccines has contributed to our understanding and offered insights into addressing undervaccination in specific populations, including Native Americans. Historical factors and vaccine hesitancy should be considered. The distribution of vaccines in the US should prioritize the needs of endangered spiritual communities, pregnant women, native people, and those previously excluded from clinical trials [73, 74]. The combination of cancer vaccines and therapeutic approaches has been explored to enhance immunogenic modulation. Some combinations show promise compared to historical data but may not meet the primary endpoint criteria of clinical trials. Studies with negative outcomes highlight the complexity of developing effective cancer vaccines [75]. However, recent advancements in various scientific fields have reignited interest and progress in the development of therapeutic cancer vaccines, offering hope for improved treatment strategies [76]. For example, the Human Vaccines Project has provided insights into the roadmap for the development of new cancer vaccines. These vaccines target antigens that are believed to induce a stronger immune response against cancer cells than the conventional self-cancer antigens [74]. Historical data indicate that the majority of cancer vaccine trials have been conducted without immune checkpoint blockade, and limited clinical activity has been observed thus far [75]. The neoantigen approach did not yield desired cancer vaccine targets. However, certain vaccines have shown resistance to host tumor-associated antigens, suggesting their potential clinical utility [76]. The development of a successful vaccine highlights successes and failures encountered, emphasizing the need for ongoing studies and innovations to improve patient outcomes and combat this disease effectively [77].

### 2.1 Lessons Learned: The Journey of Tumor Heterogeneity: An Inside Look

This emphasizes the importance of previous experiences in vaccine development for future strategies as well as the contributions of advocacy groups, mobilization efforts, and the successful introduction of HPV vaccines in low- and middle-income countries [78]. Combating the immune evasion strategies of cancer cells is crucial for vaccine development. An important approach to restoring cancer vaccine effectiveness is using immune checkpoint inhibitors, which counteract these evasion strategies. Cancer cells employ various methods to evade immune destruction, including resistance to T cell killing and cytokines, immune escape through antigen release, packaging, proliferation, evasion of immune recognition, and immune infiltration [79-81]. These strategies highlight the complexity of cancer efforts to evade the immune response. Another study demonstrated that low major histocompatibility complex (MHC) class I proteins are expressed on the surface of nucleated cells and present intracellular antigens to immune cells, allowing the immune system to detect foreign or abnormal proteins [82]. Another well-established mechanism of immune evasion in tumor development is the involvement of myeloid-derived suppressor cells, which contribute to immune evasion through myelopoiesis [83], indicating the diversity of evasion mechanisms. Furthermore, immune gene mutations, including JAK1, intensify immune evasion in colorectal cancer with microsatellite instability [84]. Combining cancer vaccines with other immunotherapies, like immune checkpoint inhibitors, may enhance immune evasion mechanisms. Targeted immune checkpoint inhibitors have been developed to disrupt pathways exploited by cancer cells to evade immune surveillance. These inhibitors can block inhibitory immune checkpoint pathways, enhancing cancer vaccine activity and enabling full functioning of the innate immune response. This leads to the targeting and destruction of cancer cells [79, 85-87]. Fig. (**7**) is a network visualization highlighting interconnected pathways and nodes of interest in modulating immune responses, with particular emphasis on PD-L1 and associated signaling components.

## 3 TYPES OF CANCER VACCINES

Cancer vaccines aim to stimulate the immune system to attack the cancer cells. They can be categorized based on their mechanisms and components, such as peptides, whole cells, nucleic acid-based vaccines, and neoantigen vaccines [88]. Peptide vaccines contain epitopes of antigen proteins or fragments bound to the major histocompatibility complex of tumor-associated antigens. These peptides trigger an immune response, leading to the recognition and elimination of cancer cells [89]. Another type of nucleic acid vaccine is mRNA, which provides genetic information to the host cells, enabling them to produce a specific antigen. This allows the immune system to mount a targeted immune response against tumors [89]. Neoantigen vaccines have recently emerged as promising approaches in the field of cancer vaccines [88].

These vaccines work by identifying neoantigens from tumor-specific mutations and personalizing the neoantigen vaccine approach for each patient based on genetic mutations, as shown in Table **3**, eliciting an immune response against the target and clearing cancerous cells. HPV vaccination is recommended as a primary prevention method for HPV-related cancers, such as cervical cancer and genital warts, in adolescent females [90, 91]. The bivalent vaccine offers protection against HPV-16 and HPV-18, whereas the quadrivalent vaccine prevents HPV-6 and HPV-11 [91]. These strategies for harnessing the immune system to recognize and eradicate cancer cells include peptide-, cell-, and nucleic acid-based neoantigen vaccines. Neoantigens and HPV vaccines are outstanding examples of tailored treatments likely to revolutionize preventive and therapeutic efforts against cancer.

### 3.1 Preventive Cancer Vaccines (PCV)

The development of cancer prevention vaccines has emerged as a promising strategy for treatment. The success of mRNA vaccines against COVID-19 has paved the way for mRNA-based cancer vaccines, showing promising results in clinical trials of advanced solid tumors [18]. Prophylactic vaccines, also known as preventive vaccines, have been designed to prevent cancer (Fig. **8**). They function by immunizing against infectious agents that cause certain cancers. These vaccines are administered to individuals who are unaffected by cancer. The most well-known examples are HPV and hepatitis B vaccines [92].

### 3.2 Human Papillomavirus (HPV) Vaccine: A Triumph in Cancer Prevention

The World Health Organization (WHO) recommends vaccination to reduce the number of cervical cancer cases caused by HPV. Vaccines should be administered before sexual activity begins [93]. It is highly effective against cervical cancer associated with HPV types 16 and 18, when administered before exposure. HPV vaccination is also effective at preventing other HPV-related cancers [94]. Therapeutic cancer vaccines have been developed to boost a patient's immune response and eliminate cancer cells [95]. These vaccines have been used to treat and prevent various diseases [96]. Peptide vaccines have shown significant anticancer effects, leading to improved survival rates in most cases [97].

HPV vaccines have been reported to prevent cancer development in parts of the female body other than the cervix and in men, such as the anus and throat. Physicians recognize that HPV vaccination is an ideal way to prevent many anogenital cancers, particularly cervical, vaginal, and vulvar carcinomas, which can be fatal [98]. However, prophylactic HPV vaccination has also been shown to prevent oropharyngeal cancer. In this respect, studies have revealed that prophylactic HPV vaccines can reduce the incidence of this type of neoplasm [99]. The administration of a quadrivalent vaccine against cervical cancer in men underlines the need to vaccinate males to prevent cancer. Vaccinating men protects them against various forms of cancer that emanate from HPV infection [100]. Researchers are currently working on therapeutic HPV vaccination approaches against cervical cancer, to illustrate the shift in vaccine application from preventing the use of vaccines in treatment protocols. This indicates the huge impact of HPV vaccines on cancer prevention and control [101]. This reinforces the success and potential of vaccines to reduce the burden of HPV-related cancers worldwide. HPV is the primary cause of various cancers in various locations in the body, and much attention has been paid to its association with cervical cancer. Vaccines such as Gardasil and Cervarix prevent many major cancers, including cervical and some breast cancers, in vaccinated individuals [102]. These vaccines induce the production of HPV antibodies due to the presence of virus-like particles that resemble viruses. This vaccine showed maximum protection against infection if administered before HPV exposure [95]. Vaccines such as Gardasil and Cervarix are crucial in reducing the incidence of various significant cancers, such as cervical and certain types of breast cancer, in vaccinated individuals [103, 104]. Vaccination has been associated with a reduced risk of HPV-related cancers and provides a holistic approach to cancer prevention [105].

### 3.3 Hepatitis B Vaccine (HBV): A Shield Against Hepatocellular Carcinoma

A hepatitis B vaccine is necessary to prevent Hepatocellular Carcinoma (HCC), a common primary liver cancer caused by chronic HBV infection. Hosaka *et al.* proved that long-term entecavir treatment in 2013 reduced the incidence of HCC in HBV-infected patients compared to other medications [106]. Universal vaccination programs in Taiwan can reduce the incidence by 70-90%. Long-term follow-up is necessary to determine the effect of mass vaccination on HCC rates. Chronic HBV infection is a major risk factor for HCC [107]. This is because of the illustrative processes of liver cirrhosis and cancer [108]. The recommended vaccines, vaccines B and B, and the Vaccines have been associated with a reduced prevalence of hepatocellular carcinoma in vaccinated subjects [109]. These vaccines trigger an antibody response to the virus and, if administered before exposure to HBV, offer protection [110].

Indeed, a considerable proportion of HCC cases have recently been attributed to HBV infection, more precisely in countries such as China and Korea, both endemic to chronic HBV infection. Cirrhosis also plays a significant role in the development of HBV [111, 112]. Antiviral treatment with nucleoside analogs reduces the risk of HCC development in patients with chronic HBV infection. This is the foundation of antiviral therapy and vaccination against HCC [113, 114]. In summary, the Hepatitis B vaccine confers protection against HCC by preventing chronic HBV infection, an etiological risk factor for aggressive liver cancer. This is essential for countries that can reduce the global burden of HBV-related HCC through vaccination and antiviral therapies.

## 4 THERAPEUTIC CANCER VACCINES

Therapeutic cancer vaccines are a novel cancer treatment. This study aimed to use the patient’s immune system to detect abnormal cells. However, clinical efficacy remains a challenge. Recently, combining them with other immunotherapies or targeted therapies has been explored for better effectiveness [115]. Sipuleucel-T was the first vaccine approved by the FDA for the treatment of metastatic castrate-resistant prostate cancer [116]. This was a milestone in the field. Outer membrane vesicles (OMVs) are nanosized extracellular vesicles derived from gram-negative bacteria that are known for their strong immunogenic potential in cancer vaccine development. These vaccines offer safe and wide application prospects as new tools for cancer treatment [117]. They function by expressing virus-like particles, and their structure is similar to that of cancer cells. This, in turn, provokes an antibody response against the targeted tumor antigens [118]. Therefore, one of the central aims of therapeutic cancer vaccines is to elicit tumor-specific immune responses. Coding sequences (CDS) are defined regions of DNA or RNA that encode functional proteins and play a critical role in improving antigen presentation efficiency in vaccine design [118].

Unlike preventive vaccines, therapeutic cancer vaccines are administered specifically to cancer patients to enhance their immune response, particularly the CD8^+^ T-cell response, with the help of effective adjuvants [119]. Targeting neoantigens using personalized RNA mutanome- and peptide-based vaccines can lead to therapeutic immunity against cancer [119, 120]. Despite the challenges associated with these vaccines, they continue to be actively researched owing to their potential clinical application [121]. In conclusion, therapeutic cancer vaccines aim to reprogram the immune system to eliminate cancer cells. Ongoing research and advancements in combination therapies will improve patient outcomes.

Sipuleucel-T processes cells obtained from the patient and exposed to a fusion protein containing prostatic acid phosphatase (PAP) and granulocyte-macrophage colony-stimulating factor (GM-CSF), activating dendritic cells and fighting cancer cells with PAP in the prostate. Clinical trials of sipuleucel-T have proven survival benefits and set a landmark in cancer immunotherapy, bringing it to the forefront and suggesting potential applications in cancer treatment [122, 123]. Experimental vaccines developed for pancreatic cancer treatment include GVAX, described below. The vaccine consists of irradiated tumor cells secreting granulocyte-macrophage colony-stimulating factor (GM-CSF). When administered, GVAX stimulates the immune system to increase the response against pancreatic tumor cells. Despite dissimilar clinical trial outcomes, this method is a significant step toward developing targeted therapeutic cancer vaccines, especially for difficult-to-treat malignancies like pancreatic carcinoma [124, 125]. The development and success of sipuleucel-T reflect the growing importance of therapeutic cancer vaccines. Continued research on vaccines such as GVAX is among the major strides in cancer immunotherapy, providing new hope for patients.

## 5 PERSONALIZED CANCER VACCINES

Personalized cancer vaccines are an emerging avenue for cancer treatment using immunotherapy. These vaccines act upon T-cells of the immunological response against tumor antigens of the patient, personalizing the treatment. Evidence suggests that neoantigen-based personalized cancer vaccines can induce a specific, target-oriented, and effective immune response against cancer, showing potency in individualized immunotherapy in cancer patients [62, 126].

Technological advances have enabled the identification of individual mutations at specific sites. More recent and sophisticated sequencing technologies have made it possible to design such cancer vaccines in a personalized manner according to the neoantigens unique to each patient's tumor [127]. Such vaccines have been very promising in clinical trials, which led to an improvement in OS and elicited potent T-cell responses against multiple tumors [128]. Moreover, mRNA vaccines are promising for the treatment of cancers, and one of their strategies has already demonstrated the effectiveness of personalized therapy in the treatment of certain types of cancers [128, 129].

Therefore, the development of personalized vaccines requires a multidisciplinary approach in disciplines such as bioinformatics, immunology, and oncology. This study aimed to develop vaccines that would target unique antigens in a patient's tumor. The use of individual mutations and tumor-specific antigens in the development of personalized cancer vaccines has been shown to elicit robust immune responses against cancer cells, thereby improving patient survival [62]. The new generation of cancer vaccines targeting genomic mutations specific to each patient's tumor is at the forefront of personalized medicine. Such vaccines include short peptides resulting from these mutations, called neoantigens, which can be found within tumors [15]. The synthesis or choice of cancer vaccines may be realized based on a patient's genome analysis or potential to elicit an immune response. These vaccines have the advantage of being highly personalized, recognizing neoantigens on cancer cells from each patient, thereby reducing side effects while maximizing therapeutic response [130]. Clinical trials for neoantigen-based vaccines are at an early stage, but are promising. They can shift cancer treatment practices by providing molecular therapies tailored to individual patients. The vaccine is derived from specific polypeptide chains of mutated proteins expressed only in tumor cells. This is a new frontier in cancer treatment due to its effectiveness and precision [88, 130]. If successfully developed, neoantigen vaccines could become important milestones in personalized medicine, providing renewed hope for more effective and targeted cancer treatments. Continuous research in this field is unfolding the transformative power of vaccines in revolutionizing cancer therapy [131]. As shown in Fig. (**9**), the gene interaction network elucidates key molecular targets for vaccine development, emphasizing tumor antigens and immune-activating nodes.

### 5.1 Cancer Vaccines: The Emerging Option for Cancer Treatment

Cancer vaccines represent an innovative approach to treating patients with advanced stages of the disease. With dissimilar modes of activity against chemotherapy and radiation therapies, cancer vaccines activate defense mechanisms to fight this disease [28]. Cancer vaccines can reduce toxicity and inhibit the development of cancer. These vaccines prevent the development of cancers caused by viruses and bacteria, thereby opening new avenues for cancer treatment. This opens the possibility of developing vaccines for cancer prevention and therapy. Further research into immunology and cancer biology could allow for the merging of different cancer vaccines with other forms of immunotherapy to provide an integrated approach to cancer immunotherapy. The ultimate goal is to improve the body's natural defenses to destroy malignant cells while limiting as little damage as possible to normal tissues [22].

## 6 MECHANISMS OF CANCER VACCINES

The immune system is a network of defense mechanisms consisting of cells and tissues that specialize in developing immunity against infections or diseases [132]. Innate and adaptive immunity. Innate immunity is the first line of defense against infection, not recognizing the pathogen but rather the general pattern of infection. The adaptive immune system is a part of the secondary defense system and is more specialized. In some respects, they can recognize and attack specific pathogens. Importantly, it is characterized by a 'memory' feature that permits a prompt and efficient response to an attack by the same pathogen once encountered [133].

Specifically, mechanisms to hide from or mislead the immune system include changes in surface proteins that prevent their recognition or induce immune suppression in the tumor microenvironment. Cancer vaccines are the cardinal components of immunotherapy because they activate the immune system to recognize and destroy cancer cells. This means that such vaccines work by counteracting the mechanism of evading the immune response through cancer cells and then exploiting these responses for recognition and elimination of abnormal or malignant cells [57].

It is an internalization and processing process that involves antigen-presenting cells. The processed antigens are then displayed with major histocompatibility complex molecules to other immune cells. MHC class I antigens recognize and present resident antigens to CD8^+^ cytotoxic T-cells. MHC class II antigens recognize and present antigens to CD4^+^ helper T cells. The latter activates CD8^+^ T cells and B cells. Meaningless antigen presentation in cancer immunology: Host-antigen presentation to the immune system; after recognition, eliminating abnormal cells, including precancerous cells [134, 135]. Normally, cancer cells downregulate MHC molecules to avoid immune surveillance using inhibitory molecules such as PD-1. Therefore, the response of immunotherapy to tumor-specific antigens should be increased. This implies that these antigens are offered to immune cells to recognize and mount an attack. Dendritic cells play a key role in presenting these antigens to T cells, thereby activating them to recognize them, allowing the latter to kill cancerous cells [136, 137].

## 7 TYPES OF CANCER VACCINES

### 7.1 Peptide-Based Vaccines

A peptide vaccine is a cancer vaccine that uses a synthetic short peptide derived from tumor antigens, as shown in Table **4**. It activates the immune system to eliminate cancer cells by stimulating an immune response against the tumor's antigenic peptides [138].

### 7.2 Dendritic Cell-based Vaccines

These immune cells, known as "dendritic cell-based vaccines," target antigenic peptides and load tumor antigens. These are administered via injections or skin-prick methods. By presenting tumor antigens, these vaccines activate T-cells to initiate an immune response [139]. Viral vector vaccines use modified viruses to deliver immune-stimulating materials, enabling the presentation of tumor antigens to immune cells, particularly dendritic cells. The dendritic cells are then activated to elicit an immune response against tumors. This approach holds promise for providing robust and targeted immunity against tumors [140].

### 7.3 mRNA Vaccines

Cancer mRNA vaccines introduce foreign mRNA into cells to trigger the immune system's recognition of tumor antigens. This recognition enables the immune system to target and eliminate cancer cells [141].

### 7.4 DNA Vaccines

DNA cancer vaccines direct cells to produce tumor antigens, stimulating the immune system to target and eliminate tumor cells, as summarized in Table **3**. Administered through injection, these vaccines provide DNA sequences encoding tumor antigens, initiating an immune response against the tumor. Cancer vaccination strategies aim to equip the immune response to identify, target, and eliminate cancer cells, proving an effective approach to cancer treatment [142].

### 7.5 How Cancer Vaccines Activate the Immune System

The main goal of cancer vaccines is to activate the immune system to attack cancer cells. The vaccine uses antigens related to tumor-associated antigens (TAAs) and tumor-specific antigens (TSAs) that suppress the immune response of the body [143]. Cancer vaccines modulate the immune system to identify and attack specific antigens present in the cancer cells. This triggers an immune response in tumors [28]. Therapeutic cancer vaccines activate the host immune system and can be potentially enhanced by other therapies [69].

These vaccines have been designed to take advantage of immunity in a manner that induces long-lasting memory against tumor cells with minimal toxicity [14]. Therapeutic cancer vaccines induce precise responses along antigen targets observed in cancer cells, targeting only antigens uniquely present within cancerous cells. These vaccines activate patients' immune systems and facilitate blood-activated CTLs' migration into tumor tissues. While monoclonal antibody treatments work, therapeutic cancer vaccines ensure an immune response specific to lymph nodes, increasing activated CTLs at the tumor site [144]. Furthermore, cancer vaccines activate antigen-specific adaptive responses of host immunization to facilitate antibody production and T-cell proliferation [145]. Cancer vaccines enhance the immune system by expressing tumor-derived antigens to cause an antibody response against these antigens within cancerous cells, leading to activated immune cells moving into tumor-infected tissues. Cancer vaccines have enormous potential as therapies because they can harness the immune system's intrinsic ability to recognize and destroy cancer cells (Table **5**).

### 7.6 How Cancer Vaccines Target and Destroy Cancer Cells

Activated T cells are now ready to carry out their multifaceted counterattack, specifically targeting and ultimately eliminating cancer cells [146]. Cytotoxic T cells, also called CD8+ T cell effector cells, act on cancer cells by releasing cytotoxic molecules like perforins and granzymes. These molecules induce apoptosis in tumor cells [147]. In addition, T cells collaborate with other immune cells to enhance the immune response against cancer. Other vaccines have been designed to counter the evasion tactics employed by cancer cells, as shown in Fig. (**10**). These vaccines boost antibody production and strengthen the immune system by blocking immune checkpoints [148]. The immune system's control processes counteract excessive immune activity; yet, cancer cells can exploit these to escape detection. T cells cooperate with other immune cells to augment the response against cancer. Some vaccines counter evasion tactics used by cancer cells, enhancing antibody production and fortifying the immune system by obstructing checkpoints. Cancer vaccines also modify the tumor microenvironment, making it more vulnerable to clearance. This may enhance immune cell infiltration into tumors and diminish immunosuppressive factors [28]. Consequently, immune surveillance is heightened, enabling the identification and eradication of cancer cells at initial locations. Cancer vaccines also stimulate memory T cells that persist long after the initial immune response. During relapse, memory T cells can recognize and target cancer cells. This means immune assault on cancer cells is rapidly initiated and effective [149, 150].

Cancer vaccines should follow cancer immunology principles and leverage immune responses against cancer cells (Table **6**). Peptide vaccines can significantly enhance the immune system's ability to recognize and combat cancer cell evasion mechanisms, offering a novel approach to treating a previously incurable condition.

### 7.7 Current State of Cancer Vaccines

FDA-approved cancer vaccines represent a breakthrough in oncology and a new era in cancer treatment and immunotherapy [151]. Provenge targets metastatic castration-resistant prostate cancer, Gardasil 9 protects against certain cancers linked to HPV, T-VEC triggers an immune response against melanoma, Heplisav-B is prophylactic against hepatitis B, and Bexsero is approved because of its potential effectiveness in preventing glioblastoma and other aggressive brain cancers (Table **7**) [27, 152]. This approval bridged the gap between cancer prevention and treatment.

### 7.8 Limitations and Side Effects

These limitations and practical considerations in clinical application have hindered the use of cancer vaccines as therapeutic and prophylactic agents.

1. Limited Applicability: Most cancer vaccines target specific species, organs, genders, and age groups, limiting their wider applicability. For instance, sipuleucel-T is approved for castration-resistant prostate cancer, Gardasil 9 prevents HPV-related cancers, and Imlygic treats recurrent stages 1c-2c [28, 132].

2. Variable Response: Patient responses to cancer vaccines vary over time and are influenced by factors such as vaccine characteristics, immune system status, and cancer type [153].

3. Additional adverse events: Similar to any medical intervention, cancer vaccines have associated adverse effects or side effects. Although rare, local reactions and flu-like symptoms [22].

4. Ethical Considerations: Personalized cancer vaccines that target patient-specific neoantigens raise concerns regarding their fair distribution and accessibility to healthcare [154].

5. Cost: The research and development of cancer vaccines require significant financial resources, which may render them too expensive or unaffordable for healthcare systems.

6. Sample Size: Cancer vaccines require a large number of subjects in many clinical trials, which might be costly to handle and maintain resources [155].

7. Heterogeneity: Cancer heterogeneity leads to variations in vaccine responses among patients; hence, it is difficult to predict an effective immune response in these patients [156].

8. Immunosuppression: Suppression of the immune system in the later stages of a disease weakens the potency of cancer vaccines. Cocktail therapies, including immune checkpoint inhibitors and other immune modulators, are being evaluated for these anomalies [157].

9. Long-term monitoring was performed to determine the protection provided by these vaccines and the possible adverse effects [158].

These obstacles warrant careful study to help realize the benefits of cancer vaccines and be appropriately sensitive to the complexities of their use. However, such a future must centrally focus on ethics.

### 7.9 Promising Antigens and Targets

An effective cancer vaccine requires the selection of appropriate antigens or targets that elicit a strong immune response against cancer cells, without harming normal tissues [159]. Recent advances in cancer research have elucidated promising antigens and targets that may facilitate the development of efficacious cancer vaccines. Consequently, further progress in this field underscores the significance of the selection process in the design of successful cancer immunotherapies [27, 40, 122, 123, 142].

### 7.10 Identifying Cancer-Specific Antigens

One of the main challenges in the development of cancer vaccines is the identification of cancer-specific antigens. Commonly expressed antigens may cause autoimmune and undesirable side effects [89]. Recent discoveries have iden-tified antigens exclusively expressed by cancer cells, including tumor-associated antigens (TAA), which are overexpressed or abnormally expressed compared to healthy cells (Table **7**). Examples of TAA include HER2 in breast cancer and PSA in prostate cancer. Neoantigens, in contrast, are new antigens resulting from cancer cell mutations [35, 160]. They are not recognized as foreign by the immune system. Advances in genomics and computation have improved neoantigen detection, making it a desirable target for personalized cancer vaccines. Cancer testis antigens (CTAs), primarily found in germ cells but re-expressed in neoplastic cells, show promise as candidates for cancer vaccines due to limited expression in normal tissues [161]. This approach offers the possibility of treatment with minimal side effects on normal tissues. Extensive research is underway on these cancer-specific antigens to develop safe and effective cancer vaccines, as summarized in Table **8**, paving the way for future cancer immunotherapies [162].

### 7.11 Promising Tumor-Associated Antigens and Neoantigens

Tumor-associated antigens and neoantigens are key targets for anti-tumor vaccine development. These antigens have received significant attention in cancer treatment. An innovative approach involves creating personalized neoantigens for individual patients. These vaccines aim to stimulate an immune response against neoantigens specific to the patient's tumor, offering new treatment opportunities. The MAGE-A family represents a promising target for developing antigenic vaccines against melanoma [155]. Antigens of this family are highly immunogenic and selectively expressed in certain tumors, making them ideal targets with enormous potential. Research studies targeting common KRAS mutations in sporadic CRC and other cancers, including IDH mutations in gliomas and AML, are underway [163]. Interestingly, these mutations yield neoantigens, which are good targets for vaccination strategies against AML. Neo-antigen and personalized cancer vaccines are new frontiers in cancer immunotherapy.

These vaccines have the advantage of eliciting a neo-antigen-specific immune response and reducing potential off-target effects, which could cause autoimmune reactions. This design makes them easily adaptable to changing neoantigen landscapes. Early reports indicate that neo-antigen vaccines show promise toward positive clinical outcomes, not just from eliciting a favorable immune response but also as an indicator of improved therapeutic effectiveness [131, 164]. Combinations of individualized onco-vaccines with other immunotherapies, like immune checkpoint inhibitors, are likely to enhance immune responses and provide better clinical advantages. In summary, identifying the most promising tumor-associated neoantigens in cancer research provides insight into the future of personalized cancer vaccines as a stepping stone for treating different cancer types [14, 56, 165].

## 8 IMMUNOTHERAPY COMBINATIONS

### 8.1 Synergy between Cancer Vaccines and Checkpoint Inhibitors

A better understanding of the immune system's role in both destroying and developing malignant cells has led to new cancer immunotherapy strategies, especially vaccines and checkpoint inhibitors [17, 20]. Cancer vaccines are designed to elicit immune responses against specific cancers and neoantigens. However, tumors often establish a suppressive environment that compromises immune cell functionality [88]. Checkpoint inhibitors, such as PD-1 or CTLA-4, function by abolishing the immune brakes employed by tumors to evade immune recognition. These inhibitors rejuvenate the immune cells and augment their capacity to target cancer cells [115, 161, 162]. The interplay between cancer vaccines and checkpoint inhibitors is believed to have a reciprocal effect on the immune response. Although cancer vaccines prime immune cells to identify cancer-specific antigens, checkpoint inhibitors release constraints on the immune response, enabling them to discern and attack cancer cells more accurately, as shown in Table **9**. This combination promotes a profound and sustained anti-tumor immune response, presenting promising prospects for cancer therapy [17, 115, 121].

### 8.2 Clinical Trials and Outcomes of Combination Therapies

Clinical data have demonstrated that the combination of vaccines with checkpoint inhibitors is effective against several types of cancer [166, 167]. Several trials have shown that adding the anti-CTLA-4 checkpoint inhibitor ipilimumab to the cancer vaccine GVAX in patients with advanced-stage melanoma yielded similar overall survival benefits as chemotherapy. Similar results were observed with the combination of the cancer vaccine talimogene laherparepvec (T-VEC) with pembrolizumab [68]. Combining immunotherapy with checkpoint inhibitors significantly increases the response rates in non-small cell lung cancer. Notable combination trials include nivolumab/sumab and sumab (anti-PD-1) in conjunction with the cancer vaccine MAGE-A3, which are highly anticipated in this field [67]. A randomized trial involving men who received the cancer vaccine PROSTVAC in combination with ipilimumab also demonstrated an improved overall survival [168]. This combination approach is under clinical investigat- ion for breast and ovarian cancers, as well as in an ongoing trial for glioblastoma [169]. Although both combination treatments are quite promising in terms of clinical results, they also have some possible disadvantages and toxicities. Nonetheless, they do bring with them the efficacy signal of survival advantage and increased response rates for various other types of cancer [170]. The increased immune response due to immunotherapy is associated with additional risks for autoimmune reactions; hence, regular follow-up of patients is required [171]. Nevertheless, one challenge remains the identification of predictive biomarkers for treatment response and the patient population that will derive the greatest benefit from these combination approaches [172]. Further research is needed to determine the optimal sequencing and timing of cancer vaccines and checkpoint inhibitor administration to achieve additive effects without compromising patient well-being [173]. The affordability of combination drugs may pose a barrier if their prices rise to the point where they become inaccessible to certain patients in need [174]. Given the complex and highly heterogeneous nature of tumors, it is challenging to develop combinations that effectively target all tumor characteristics [175]. The integration of cancer vaccines with checkpoint inhibitors holds immense promise for enhancing anti-tumor immunity. However, further research and integration of such combination immunotherapies with standard cancer treatment regimens are eagerly awaited, as they have the potential to significantly improve patient outcomes [28]. Crucially, the development of these technologies has contributed to the emergence of therapeutically relevant cancer vaccines, offering new hopes for improved therapeutic options against cancer as shown in Table **10**.

### 8.3 Overview of Emerging Technologies and Their Potential Applications in Cancer Vaccine Development

This marked the beginning of RNA vaccines, a significant advancement in vaccine technology. Initially used for cancer immunotherapy, these vaccines have been extensively studied to combat other infectious diseases, including COVID-19 and influenza [176]. However, its most promising applications are in cancer treatment. In cancer immunotherapy, patients receive artificially synthesized mRNA strands derived from RNA vaccines. When translated within the recipient's cytoplasm, these mRNA molecules generate antigens, provoking an immune response against antigenic determinants on cancer cell surfaces [18]. Additionally, the design and development of RNA vaccines are less complex than traditional vaccines, which is highly beneficial in the field of oncology, where swift treatment design is often crucial [141]. By contrast, viral vector vaccines integrate genetic material into the host genome, thereby enhancing their safety [52]. Collectively, RNA-based vaccines represent a significant revolution in personalized, rapid, and effective immuno-oncology by harnessing the body's inherent immune defense mechanisms.

### 8.4 Nanotechnology in Vaccine Delivery

Nanotechnology has potential for drug delivery, especially in cancer vaccine development. It enables engineered nanoparticle vaccines to enhance antigen adjuvant delivery through focused delivery and improve immune response. These nanobiles may be designed to increase specificity for immune or tumor cells, reducing off-target effects [177, 178]. Artificial intelligence is an underpinning component in rational vaccine design, with machine learning playing a significant role. AI has expedited antigen discovery and immune responses, shortening the testing process. It helps predict antigens or neoantigens for cancer vaccines by identifying tumor-specific molecules. Modeling and predicting immune responses will aid in designing vaccines that provide strong, long-lasting immunity [179]. AI optimization improves decision-making on adjuvants, dosing, and administration. Using AI with molecular and clinical trial data can predict patient subgroups who will react well to certain vaccines or therapies. This improves trial outcomes and efficiency of cancer treatment trials [180].

## 9 PRODRUG CANCER THERAPY

Prodrug therapy comprises prodrugs that are known to have no biological activity. Certain compounds that are originally inert are released into active drugs from prodrugs that are activated by metabolic processes in the body. Therefore, innovative solutions have been developed to improve the selectivity and efficiency of prodrug-based cancer therapies. The *in vivo* effectiveness of such strategies includes prodrugs that, upon activation by oxygen radicals that are usually found in copious amounts within cancerous cells, form very reactive compounds that are toxic to many molecules, including DNA and proteins. As such, this strategy is often applied in the treatment of various cancers [181]. Another promising therapeutic strategy is prodrug self-assembled nanoparticle-bearing therapeutics because of the combined advantages of the prodrug strategy and nanocarriers [182].

Furthermore, the development of far-red light-activatable prodrugs has demonstrated their ability to efficiently eliminate cancer cells through a combination of photodynamic therapy (PDT) and site-specific chemotherapy [183]. Other strategies involve using prodrug-activating enzymes, such as antibody-directed enzyme prodrug therapy (ADEPT) and gene-directed enzyme prodrug therapy (GDEPT), which are designed to target tumor-specific antigens and transcription factors. These strategies have been investigated for use in gene cancer therapy and have shown potential for treating brain tumors [184]. Moreover, researchers have exploited mitochondria within cancer cells as targets for developing cancer-specific prodrugs [185]. Recent studies have also discussed the development of prodrug nanoparticles for cancer therapy [186]. Prodrug-based cancer therapy is a rapidly evolving field that has advanced in line with improvements in activation mechanisms, nanocarrier systems, and targeted drug delivery. These innovative strategies hold great promise for enhancing the selectivity and effectiveness of anticancer treatments while reducing off-target side effects, thus offering a promising future for cancer therapy.

## 10 CHALLENGES AND FUTURE DIRECTIONS

Many adaptive strategies are overly complex in vaccine design against cancer. Nature's complexity and genetic/phenotypic heterogeneity complicate developing universally applicable vaccines. Tumors can evade immune responses by downregulating immune checkpoint molecules, secreting immunosuppressive factors, and altering antigen presentation. Challenges include choosing immunogenic antigens and breaking immune tolerance against self-antigens in cancer cells [156, 175]. However, several emerging techniques are promising:

1. Personalized vaccines: This type of vaccine targets neoantigens specific to each patient and effectively eliminates tumor cells that have evaded the immune system. This approach utilizes advancements in genomics and bioinformatics to identify patient-specific antigens [15, 56, 127, 129, 143].

2. Combination therapy: Combining cancer vaccines with other immunotherapies, such as checkpoint inhibitors and immune system modulators, can synergistically enhance the efficacy of cancer treatments by influencing the immune system [17, 20, 68, 69, 75].

3. Immune checkpoint blockade: Targeting immunosuppressive factors secreted by tumors or immune checkpoint molecules can help overcome immune evasion [17, 121].

4. Enhanced antigen presentation: Strategies that enhance antigen presentation, such as activated dendritic cells, show promise for improving the overall efficiency of cancer vaccines [134].

5. Adjuvant innovation: Developing stronger and more targeted adjuvants can enhance the efficacy of vaccines [88].

6. Machine Learning and AI: Machine learning and artificial intelligence play crucial roles in analyzing big data within AI-driven platforms for vaccine development. These technologies enable antigen discovery, prediction of patient responses, and optimization of vaccine formulations, leading to the development of more precise cancer vaccines [33, 49, 127, 149]. These strategies and technologies offer new hope for cancer treatment by addressing barriers to cancer vaccine development, as shown in Table **11**.

## 10.1 Future of Cancer Vaccines and Integration into Standard Cancer Care

Research on cancer vaccine futures shows promise in addressing challenges. Personalized cancer vaccines elicit responses against antigens unique to each patient. These vaccines are administered with immunoregulatory agents for cancer treatment [21]. They will also play a crucial role in preventing infection by cancer-causing viruses such as HPV and hepatitis B. Furthermore, ongoing research is shedding light on how cancer vaccines can intervene in the early stages of cancer, thereby reducing recurrence rates and improving overall survival [32]. The clinical development of cancer vaccines as an integral component of cancer therapy will continue, emphasizing close monitoring of safety and effectiveness. Several obstacles must be addressed, including guidelines for vaccine administration, identification of suitable patient populations, and establishment of robust monitoring protocols [23]. Consequently, the global distribution of these vaccines is intricate, necessitating the establishment of appropriate infrastructure and distribution mechanisms. Despite these challenges, the constant pursuit of research and innovation indicates that several obstacles can be overcome in the future. Cancer vaccines, together with other modalities such as gene therapy, have the potential to become indispensable tools in the fight against cancer [187].

## 10.2 Ethical and Regulatory Considerations

Further development of cancer vaccines depends on advancements in bioethics and clinical practice. Ethical standards ensure research is conducted ethically, protecting patients' rights and well-being. Informed consent provides subjects with clear, complete, and unbiased information about the study's purpose, potential risks, benefits, and alternatives, ensuring voluntary participation based on informed choice [21]. Ethical considerations arise when weighing the potential risks to subjects against the benefits of a cancer vaccine trial. Researchers must minimize risks to subjects while maintaining the scientific value of their research [188]. Special considerations are needed for vulnerable populations like pediatric patients or those with cognitive impairment. When asking about race, it is crucial to include people from ethnic minorities [189]. Pre-informed consent should be obtained to address common therapeutic misconceptions in which participants may believe that trial enrolment guarantees benefits. The experimental nature of the trial must be emphasized for a clear understanding of therapeutic benefit [190]. Ethical concerns also involve ensuring equal access to potentially promising cancer vaccines. "The Imperatives of Justice" demand equal opportunities for participation for all eligible subjects. Overall, the development of cancer vaccines ensures compliance with ethical requirements, protecting the dignity of research and the rights and welfare of participants [191].

Ethical considerations arise when cancer vaccine trials use placebo controls due to available alternative treatments. This approach balances scientific rigor and patient well-being. In some cases, placebo controls may bring ethical advantages by ensuring all participants receive established treatments [192]. The regulatory approval of cancer vaccines follows a defined process to ensure safety and efficacy. Regulatory bodies like the European Medicines Agency (EMA) and the Food and Drug Administration (FDA) in the United States adopt a stepwise approach starting with preclinical testing, including laboratory and animal studies, before progressing to human clinical trials [193]. Clinical trials are conducted in four phases: dose and safety testing, efficacy assessment in a large cohort, ongoing safety monitoring, and benefit-risk evaluation based on preclinical studies, clinical trials, and quality control data [194]. Transparency and safety are crucial in vaccine research. Vigilant monitoring ensures accurate reporting of findings. Clinical trial data should be publicly available for independent scrutiny. Study protocols were rigorously reviewed, and data were continuously monitored. Institutional review boards and ethics committees oversee human subject protection and establish research prioritization criteria. Patient advocacy groups, communities, and stakeholders safeguard participants' rights and well-being. The regulatory processes governing cancer vaccines are comprehensive, encompassing preclinical testing as shown in Table **12**, clinical trials, and post-marketing surveillance. Sustaining transparency, adhering to safety standards, and prioritizing patient welfare are essential for cancer vaccine development [195, 196].

## CONCLUSION

Advancements in cancer vaccines mark a major step forward in the field of immunotherapy, offering novel avenues to harness the immune system in combating malignant diseases. Cancer vaccines fall into three main categories: preventive, therapeutic, and personalized, each employing distinct mechanisms to enhance immune recognition and facilitate the elimination of cancer cells. Promising results have emerged from recent clinical trials, with several vaccines already approved for clinical use, thereby paving the way for continued innovation and refinement.

When combined with other immunotherapeutic agents, such as immune checkpoint inhibitors, cancer vaccines have demonstrated improved therapeutic outcomes by activating T cells and mitigating immune evasion mechanisms. Personalized cancer vaccines, which are designed to target neoantigens unique to an individual’s tumor, represent a significant advance in precision medicine by offering tailored therapeutic options based on each patient’s molecular profile.

Cutting-edge platforms, including RNA-based vaccines, viral vectors, and nanotechnology-enabled delivery systems, continue to drive progress in vaccine design, improving both immunogenicity and patient response rates. Ongoing biomarker-guided clinical trials are expected to generate critical insights into the efficacy, safety, and combinatorial potential of cancer vaccines alongside other forms of immunotherapy.

Despite this progress, challenges persist regarding equitable access, the establishment of appropriate regulatory frameworks, and the assessment of long-term efficacy. Effective translation of these scientific advances into routine clinical practice will require coordinated efforts among clinicians, researchers, and policymakers. Future investigations must prioritize overcoming access barriers, refining patient selection criteria, and integrating vaccines into existing cancer management protocols.

Ultimately, realizing the full clinical potential of cancer vaccines will depend on robust scientific evidence, sustained international collaboration, and the alignment of innovation with healthcare delivery systems. By uniting technological advancement with equitable access, cancer vaccines hold strong potential to improve patient outcomes and reshape the future of oncology."

## Figures and Tables

**Fig. (1):**
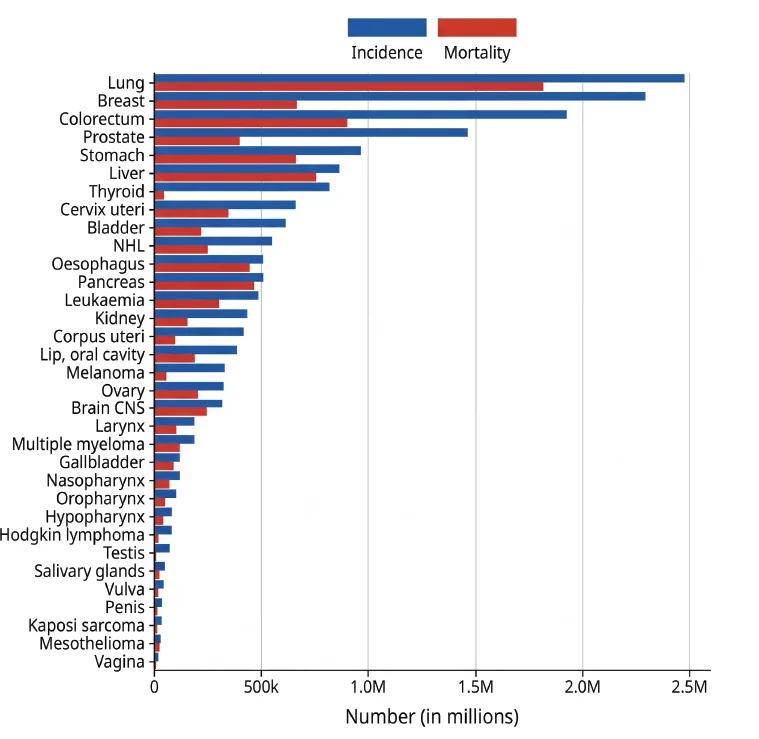
Absolute numbers of both cancer cases and deaths for both sexes in six continents, according to the World Health Organization and sourced from Cancer TODAY by the International Agency for Research on Cancer (IARC), Globocan 2022 data (accessed on 27/04.02.2024). This is based on the distribution of cancer types and their respective incidences and mortalities across populations. This kind of information is important for understanding the burden of cancer globally and the public health strategies that are required to be put in place to reduce it.

**Fig. (2):**
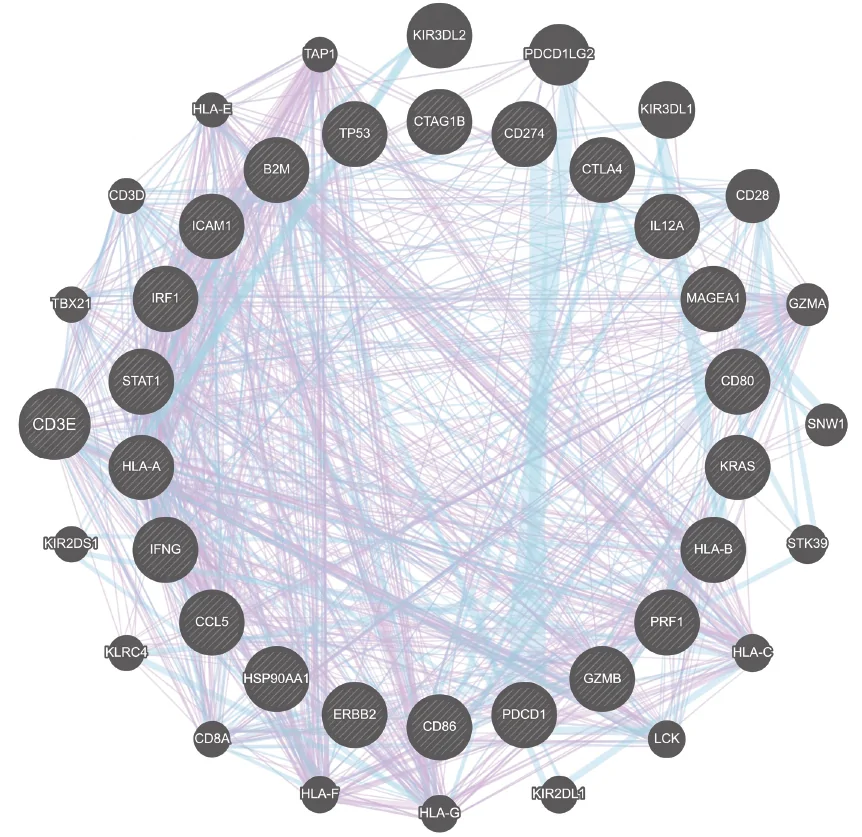
Immune-related gene interactions visualized in a circular fashion using Cystoscope and STRING. Nodes represent genes and proteins, and edges denote high-confidence interactions ≥0.7, which are supported by experimental data, text mining, and curated databases. Node size is proportional to connectivity, with highly connected nodes indicating important regulators of immune response.

**Fig. (3):**
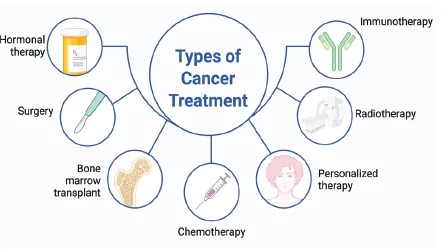
Schematic illustrations of several methods referred to as dissociation of all cancers. Treatment methods, such as immunotherapeutic agents, personalized treatments, chemotherapeutic agents, bone marrow transplant agents, and surgical and hormonal agents, differ from those used in the treatment of cancer. Each method has its own area, divergently and suitably cared for, and explains how treatment has evolved.

**Fig. (4):**
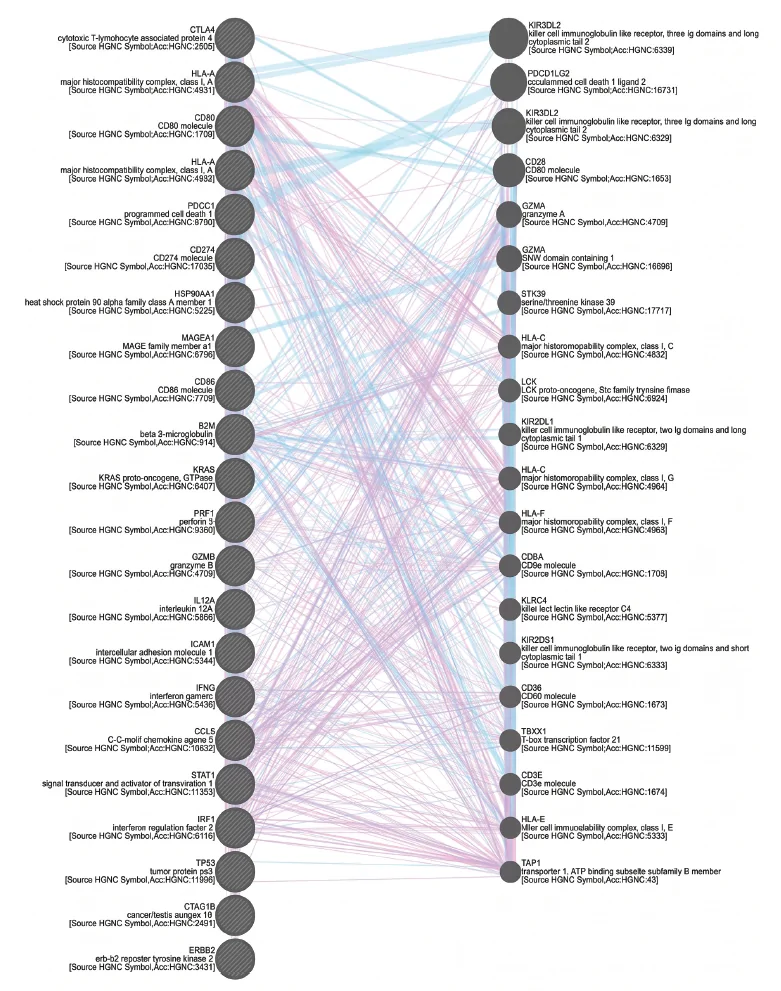
Linear map of signaling pathways associated with cancer vaccine development and immune modulation. Data were manually curated from the Kyoto Encyclopedia of Genes and Genomes (KEGG) database (pathway ID: hsa04610) and supplemented with functional interactions from STRING. The figure highlights the key proteins and signaling cascades involved in tumor immune evasion.

**Fig. (5):**
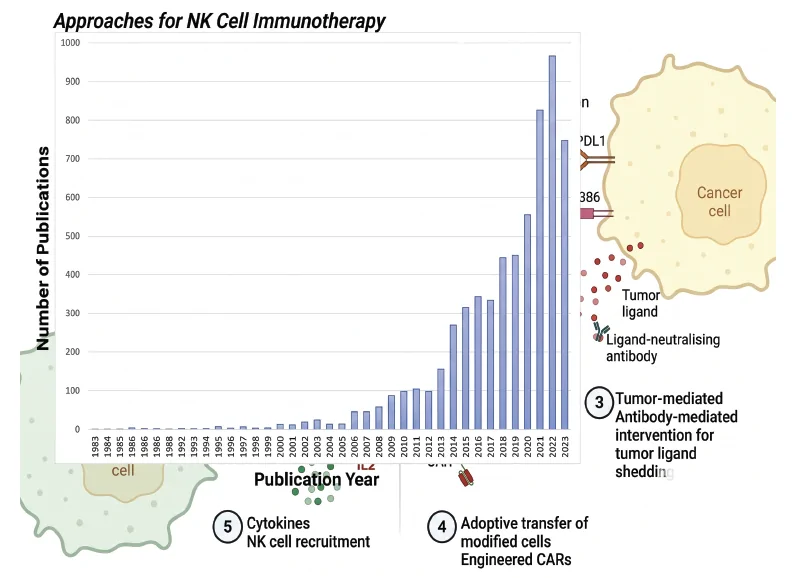
The number of publications from the PubMed database using the following keywords: “cancer vaccine publications” & “cancer vaccine research”.

**Fig. (6):**
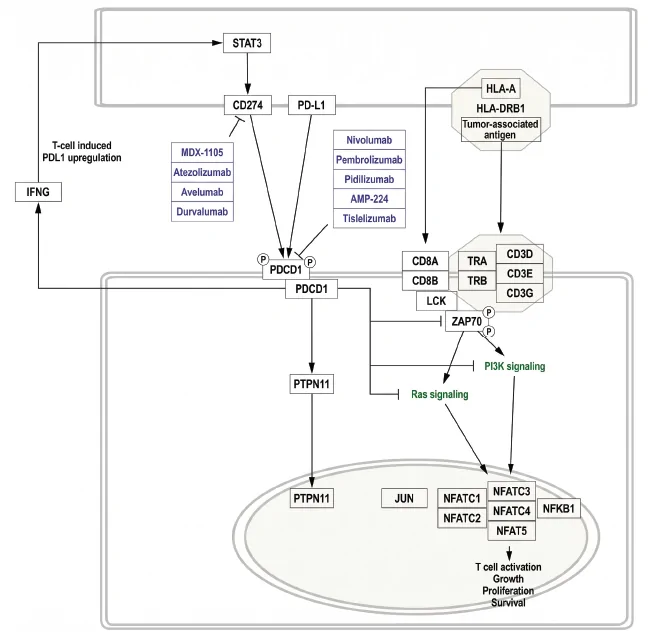
Detailed pathway map of PD-1 / PD-L1 interaction showing the role of immune evasion mechanisms. The expression of key molecules in downstream PI3K and Ras pathway signaling, leading to transcription events that regulate T cell Inhibition, is depicted: approved immune checkpoint inhibitors, among others, Nivolumab, Pembrolizumab, Atezolizumab, targeting, respectively, PD-L1-PD-1. Data extracted from KEGG: hsa04630 data were curated from KEGG pathways (ID: hsa04630) and added to recent literature to show detailed pathway maps and the role of PD-1-PD-L1 interactions in various immune evasion mechanisms.

**Fig. (7):**
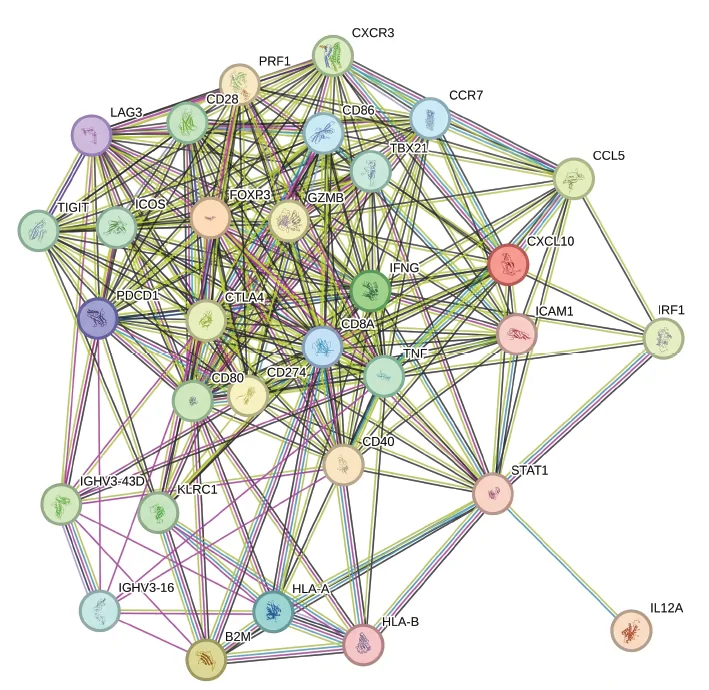
Immune response and tumor-immune interactions through the protein-protein interaction network focused on PD-L1 and associated signaling. Interactions were retrieved from the STRING database by applying a high-confidence score cutoff of ≥ 0.7. Edges represent various types of physical and functional associations that have been validated experimentally, computationally, or curated from databases.

**Fig. (8):**
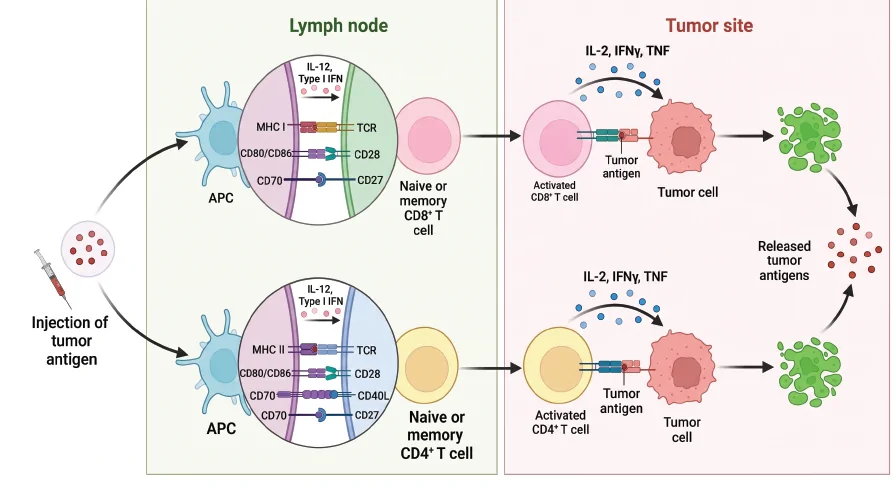
The injection specifically targets antigen-presenting cells (APCs), which can activate native or memory CD8+ T cells. Once activated, these T cells are recruited to the tumor site, where they identify the tumor cells and secrete cytokines (IL-2, IFN-γ, and TNF) that stimulate the release of tumor antigens and their subsequent destruction.

**Fig. (9):**
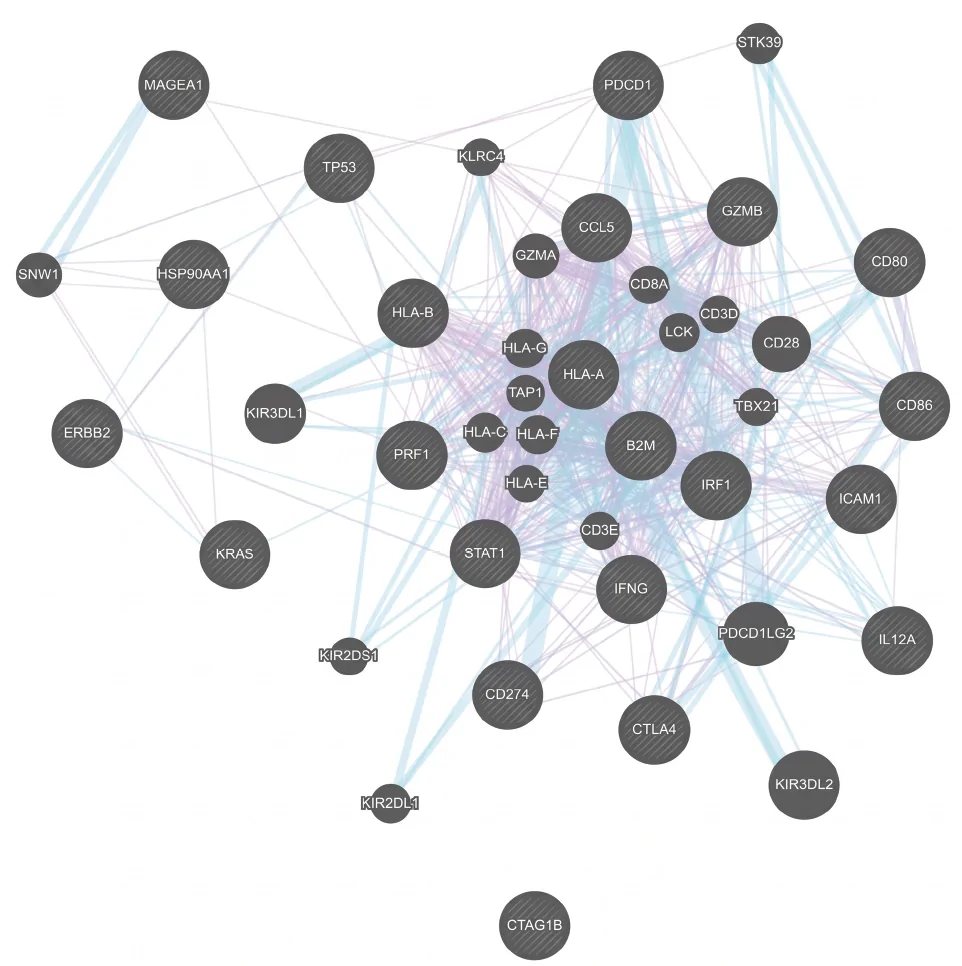
Gene interaction network identified using GeneMANIA. The central nodes represent genes encoding critical targets of immunotherapy, and the functional relationship between them was derived with the help of curated databases representing protein/physical and co-expression interactions, validated from experimental studies as well as computational predictions obtained from a curated database.

**Fig. (10):**
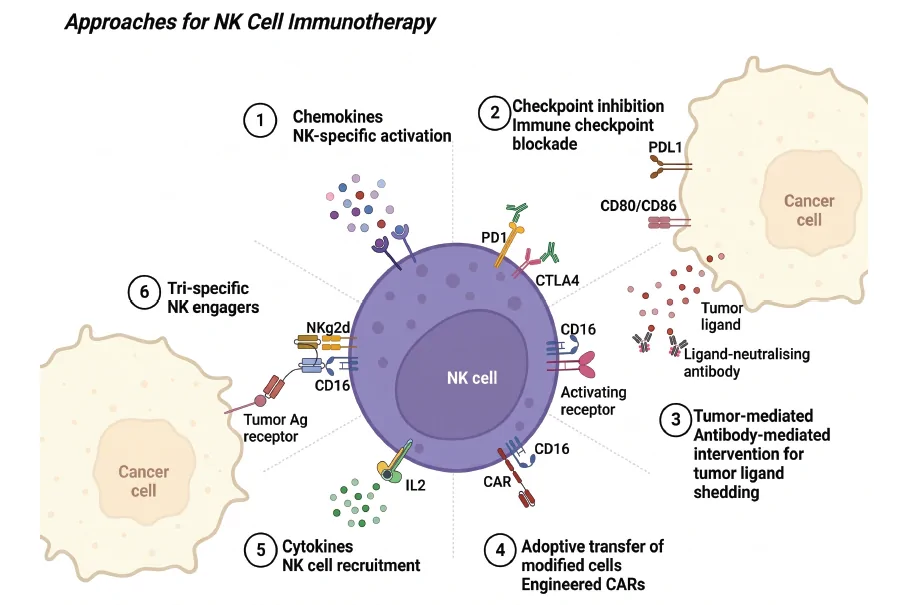
Receptors on the surface of NK cells play a crucial role in cancer immunotherapy. Receptors that are important for NK cell activation and function include IL-2, CAR, CD116, CTLA-4, and PD-1. These receptors induce various NK cell functions and enhance the anti-tumor reactivity of NK cells by interacting with ligands, such as chemokines, immune checkpoint proteins, and tumor antigens. Strategies used in cancer immunotherapy include immune checkpoint blockade, tumor-mediated antibody intervention, adoptive transfer of CAR-engineered cells, cytokine-mediated NK cell recruitment, and Tri-specific NK cell engagement.

**Table 1:** A comprehensive analysis using the SWOT framework to evaluate the benefits of cancer vaccines will expand their scope by considering their potential weaknesses, opportunities, and threats. Additionally, this analysis will include influential factors, such as public perception, competition from other established treatments, and the potential impact of these vaccines on the cancer vaccine landscape.

Aspect	Strengths	Weaknesses	Opportunities	Threats
Efficacy	-Demonstrated potential to improve clinical outcomes.	- Limited clinical success in some cancer types.	- Ongoing clinical trials refine vaccine strategies and validate efficacy.	- Competition from established cancer treatments may hinder widespread adoption.
Targeted Therapy	- Precision targeting minimizes damage to healthy tissues.	- Tumor heterogeneity challenges the identification of universal antigens.	- Ongoing research identifies novel cancer-specific antigens and neoantigens.	- Safety concerns regarding immune activation require strict monitoring, potentially leading to vaccine hesitancy.
Synergy with Other Therapies	- Enhances response rates when combined with other immunotherapies.	- Limited specificity in identifying optimal immunotherapy combinations.	- Advances in RNA vaccines, viral vectors, nanotechnology, and AI improve vaccine design.	- Complex regulatory approval processes may delay clinical implementation.
Personalization	- Enables tailored vaccines based on patient-specific antigens.	- High costs limit patient accessibility to personalized vaccines.	- Combining cancer vaccines with other immunotherapies unlocks new treatment avenues.	- Economic barriers may prevent the widespread adoption of personalized approaches.
Prevention	- Potential to reduce cancer incidence, benefiting public health.	- Lack of robust clinical data for some vaccine types.	- Global efforts aim to improve equitable access to cancer vaccines.	- Public misconceptions and vaccine hesitancy may limit acceptance.

**Table 2:** A detailed SWOT analysis of the benefits of cancer vaccines will expand their scope to include weaknesses, opportunities, and threats. This would also include influential factors, such as public perception, rivalry from other established treatment courses, and how these vaccines might affect the cancerous vaccine landscape.

Advantages of Cancer Vaccines	Strengths	Weaknesses	Opportunities	Threats
Efficacy	- Demonstrated potential to improve clinical outcomes.	- Limited clinical success in some cancer types.	- Ongoing and future clinical trials offer opportunities to refine vaccine approaches and gather more evidence of efficacy.	- Competition from established cancer treatments may hinder adoption.
Targeted Therapy	- Precision in targeting cancer cells, minimizing harm to healthy tissues.	- Challenges in addressing tumor heterogeneity with universal antigens.	- Research continues to uncover novel cancer-specific antigens and neoantigens, expanding the scope of potential targets.	- Safety concerns related to immune activation necessitate stringent monitoring and could lead to vaccine hesitancy.
Synergy with Other Therapies	- Works synergistically with other immunotherapies, improving response rates.	- Limited specificity in specifying which immunotherapies cancer vaccines work synergistically with and how this synergy improves response rates.	- Advances in RNA vaccines, viral vectors, nanotechnology, and artificial intelligence provide opportunities to enhance vaccine design and delivery.	- Rigorous regulatory approval processes can delay implementation, impacting timely patient access.
Personalization	- Tailored vaccines designed for specific patient antigens.	- Limited patient access to personalized vaccines due to cost-related limitations.	- Exploring combinations of cancer vaccines with other immunotherapies opens new avenues for treatment.	- Cost-related limitations in patient access to personalized vaccines may hinder widespread adoption.
Prevention	- Potential to reduce the incidence of certain cancers, contributing to public health.	- Lack of specific examples or clinical trial data to support this claim.	- Ongoing efforts to ensure equitable access to cancer vaccines worldwide provide opportunities for wider impact.	- Public perception and misconceptions about vaccines may affect acceptance and adoption rates.

**Table 3:** Summary of several types of cancer vaccines, their clinical stages, descriptions, actions, and examples: Each of the described categories has its advantages and challenges in the investigations made in cancer immunotherapy.

Type of Cancer Vaccine	Clinical Stage	Description	Mechanism of Action	Examples
Preventive Cancer Vaccines	Various stages	Administered before cancer exposure to prevent specific cancers.	Stimulate the immune system to recognize and destroy cancer-initiating cells or pathogens.	- HPV vaccine (Gardasil) for cervical cancer prevention. - Hepatitis B vaccine for liver cancer prevention.
Therapeutic Cancer Vaccines	Advanced stages	Designed for diagnosed cancer patients to target existing cancer cells.	Boost the immune response against cancer-specific antigens and enhance immune recognition of cancer cells.	- Sipuleucel-T (Provenge) for advanced prostate cancer. - T-VEC (Imlygic) for advanced melanoma.
Personalized Cancer Vaccines	Clinical trials	Customized for each patient based on unique tumor mutations.	Target cancer-specific neoantigens, unique to each patient's tumor, to activate the immune system.	- Individualized neoantigen vaccines (in clinical trials).
Peptide-Based Cancer Vaccines	Clinical trials	Use short protein fragments (peptides) from cancer-specific antigens.	Deliver antigenic information to the immune system and induce an immune response against cancer cells.	- GV1001 vaccine for pancreatic cancer (in clinical trials). - MAGE-A3 vaccine for melanoma and lung cancer (in clinical trials).
Dendritic Cell-Based Cancer Vaccines	Clinical use	Utilize dendritic cells to present cancer antigens to the immune system.	Isolate dendritic cells, load them with cancer antigens, and reintroduce them to activate the immune response.	- Sipuleucel-T (Provenge) for advanced prostate cancer.
Viral Vector-Based Cancer Vaccines	Clinical use	Employ modified viruses to carry cancer antigens into the body.	Viruses deliver cancer antigens to immune cells, triggering an immune response against cancer cells.	- Talimogene laherparepvec (T-VEC or Imlygic) for advanced melanoma.
RNA-Based Cancer Vaccines	Clinical trials	Use messenger RNA (mRNA) to instruct cells to produce cancer antigens.	Deliver synthetic mRNA encoding cancer antigens into cells, stimulating an immune response.	- Individualized RNA vaccines (in clinical trials for various cancer types).

**Table 4:** Mechanisms Underlying Cancer Vaccines: Molecular Processes, Clinical Relevance, and Examples.

Mechanism	Description	Molecular and Cellular Processes	Clinical Relevance	Examples
Immune Activation	Initiates an immune response against cancer cells.	- Presentation of tumor-specific antigens to cytotoxic T cells. - Activation of memory immune cells for long-term immunity.	Crucial for vaccine efficacy; stronger immune activation leads to better cancer cell destruction.	Clinical trials for various cancer types.
Antigen Presentation	Dendritic cells present cancer antigens to T cells, initiating an immune response.	- Antigen capture and processing. - Presentation on MHC molecules. - Interaction with T cells to initiate the immune response.	Essential for immune recognition of cancer cells.	Peptide-based cancer vaccines for melanoma.
Tumor-Specific Antigens	Vaccines target antigens unique to cancer cells, differentiating them from healthy cells.	- Identification of tumor-specific antigens (TSAs) or tumor-associated antigens (TAAs). - TSAs often include mutated or overexpressed proteins.	Directly correlates with vaccine specificity and effectiveness.	GV1001 vaccine for pancreatic cancer targeting RAS mutations.
Immune Memory	The immune system retains a memory of cancer antigens for future recognition.	- Long-lasting memory B cells and T cells. - Rapid response upon cancer recurrence.	Vital for sustained protection against cancer recurrence.	Therapeutic cancer vaccines like Sipuleucel-T for prostate cancer.
Adjuvants	Added to vaccines to enhance the immune response.	- Stimulation of immune cells. - Increased vaccine efficacy.	Crucial for boosting vaccine effectiveness and durability.	HPV vaccines with aluminum-based adjuvants.
Tumor Microenvironment Modulation	Some vaccines aim to modify the tumor microenvironment to make it more receptive to immune attack.	- Reduction of immunosuppressive factors. - Enhanced immune cell infiltration.	Can improve vaccine success by creating a more favorable environment for immune cells.	Oncolytic virus vaccines like T-VEC for melanoma.
Combination Therapies	Cancer vaccines are often used in combination with other immunotherapies.	- Synergy with checkpoint inhibitors. - Enhanced immune response.	Combination therapies show promise in achieving better clinical outcomes.	Clinical trials investigating the combination of cancer vaccines with checkpoint inhibitors.

**Table 5:** Mechanisms of Cancer Vaccine-Induced Immune Activation: Clinical Significance and Examples.

Antigen/Target	Description	Clinical Relevance	Personalization
Tumor-Specific Antigens (TSAs)	Unique to cancer cells, differentiating them from healthy cells.	Prime targets for cancer vaccines, offering specificity.	Involves identifying patient-specific TSAs for maximum effectiveness.
Neoantigens	Results from genomic alterations in cancer cells.	Precise targeting of cancer mutations.	Personalized neoantigen vaccines are designed based on the patient's mutation profile.
Cancer/Testis Antigens (CTAs)	Abnormally expressed in some cancers, shared among multiple types.	Potential broad-spectrum targets linked to tumor progression.	Selection of relevant CTAs tailored to the patient's cancer type and stage for increased specificity.
Oncogenic Proteins	Involved in cancer cell growth and survival.	Disrupt essential cancer pathways, often overexpressed.	Identification of dominant oncogenic proteins in the patient's cancer for targeted vaccine design.
Viral Antigens	Associated with certain virus-related cancers.	Used in cancer prevention (e.g., HPV vaccines) and treatment.	Assessment of viral infection status to select relevant viral antigens for vaccination.
Shared Tumor-Associated Antigens	Found in multiple cancer types.	Potential for broad-spectrum vaccines.	Selection of combinations of shared antigens based on the patient's cancer type to broaden vaccine applicability.

**Table 6:** Mechanisms of Cancer Vaccine-Induced Cancer Cell Destruction: Clinical Significance and Examples.

Mechanism	Description	Clinical Relevance	Examples
Antigen Recognition	Immune cells recognize cancer-specific antigens, initiating an immune response against cancer cells.	Essential for immune response targeting cancer cells.	Peptide-based vaccines for melanoma.
T Cell Activation	Activated T cells directly attack cancer cells after antigen recognition.	The effector phase of the immune response leads to cancer cell destruction.	Clinical trials combining checkpoint inhibitors with cancer vaccines.
Immune Memory	Long-term immune memory ensures a rapid response upon cancer recurrence.	Vital for sustained protection against cancer relapse and long-term immunity.	Sipuleucel-T for prostate cancer.
Cytokine Release	Immune cells release cytokines to enhance the immune response against cancer cells.	Amplifies the immune response and promotes cancer cell destruction.	Oncolytic virus vaccines like T-VEC for melanoma.
Tumor Microenvironment Modulation	Vaccines modify the tumor microenvironment to create a favorable setting for immune cell attack.	Creates an environment for efficient immune cell-mediated cancer cell destruction.	Clinical trials of dendritic cell-based vaccines in various cancer types.
Combinatorial Approaches	Combination therapies with other immunotherapies enhance immune cell-mediated cancer cell destruction.	Offers potential for improved cancer cell targeting and destruction.	Clinical trials of personalized cancer vaccines with checkpoint inhibitors.

**Table 7:** Overview of FDA-Approved Cancer Vaccines: Clinical Impact and Limitations.

Vaccine Name	Cancer Type	Antigen Target	Year of FDA Approval	Key Clinical Outcomes	Limitations and Side Effects
Gardasil 9	Cervical, HPV-related cancers	HPV types 6, 11, 16, 18, 31, 33, 45, 52, 58	2006 (Gardasil) / 2014 (Gardasil 9)	Significant reduction in HPV-related cervical cancer and genital warts.	Common side effects can include pain, swelling, and redness at the site of injection, while rare instances of severe allergic reactions have been reported.
Provenge	Prostate cancer	PAP-GM-CSF fusion protein	2010	Prolongs overall survival in metastatic prostate cancer.	Common side effects include fever, fatigue, chills, and back pain.
Sipuleucel-T	Prostate cancer	PA2024 (prostatic acid phosphatase)	2010	Improves overall survival in metastatic castration-resistant prostate cancer.	Common side effects include chills, fever, fatigue, headache, and shortness of breath.
Bacillus Calmette-Guérin (BCG)	Bladder cancer	*Mycobacterium bovis*	1990	Reduces the recurrence and progression of non-muscle-invasive bladder cancer.	Common side effects include urinary symptoms, local irritation, and occasionally flu-like symptoms.
Imlygic (T-VEC)	Melanoma	GM-CSF (Granulocyte-Macrophage Colony-Stimulating Factor)	2015	Induces regression of injected melanoma lesions and systemic antitumor effects.	Common side effects include flu-like symptoms, injection site reactions, and fatigue.
Heplisav-B	Hepatitis B	Hepatitis B surface antigen	2018	Prevents hepatitis B infection, reducing the risk of hepatocellular carcinoma.	Commonly reported adverse effects include discomfort, erythema, and edema in the vicinity of the administration site.

**Table 8:** The methods used in antigen identification for cancer vaccines can be enhanced by providing more detailed descriptions, specific examples, and discussions on future directions for each method.

Method	Description	Advantages	Challenges
Genomic Sequencing	- Analyzing cancer genomes to identify mutations that lead to neoantigen formation.	- Precision in identifying unique cancer-specific neoantigens. - Potential for personalized vaccines.	- Cost and complexity of genomic sequencing. - Variability in neoantigens among patients.
Proteomics	- Identifying antigens by studying proteins expressed in cancer cells.	- Allows the discovery of protein-based cancer-specific antigens. - May identify shared antigens.	- Limited by protein expression levels. - Proteome variability among individuals and cancer types.
RNA Sequencing	- Examining cancer cell RNA to identify transcripts encoding tumor-specific antigens.	- Offers insights into actively expressed antigens. - Potential for personalized vaccines.	- Variability in RNA expression levels. - RNA-based antigens may not always translate to protein expression.
Mass Spectrometry	- Identifying tumor-specific antigens through the analysis of peptides presented on MHC molecules.	- Direct measurement of antigen presentation. - Suitable for validation of predicted antigens.	- Requires tumor tissue or cell samples. - Limited to antigens presented on MHC molecules.
T Cell Receptor Sequencing	- Analyzing T cell receptors to identify T cells that recognize cancer-specific antigens.	- Provides insights into the immune response against cancer. - May reveal novel cancer antigens.	- Requires access to patient T cells. - Limited by T cell receptor diversity. - Challenges in identifying specific antigen recognition.
Epitope Prediction	- Computational tools predict potential antigens based on MHC binding predictions and antigen processing pathways.	- High-throughput screening for candidate antigens. - Potential for identifying shared antigens across patient cohorts.	- Predictions may yield false positives/negatives. - Validation is required to confirm antigenicity. - Limited to known epitopes.
Serological screening	- Screening patient sera against a library of tumor-associated antigens to identify antibodies that react with cancer cells.	- Relatively simple and inexpensive method. - Can identify a wide range of antigens.	- Prone to false positives. - Cannot identify new antigens.
Reverse Immunology	- Transfecting tumor cells with cDNA libraries from normal tissues, then screening for antigens recognized by patient T cells.	- Identifies new and patient-specific antigens. s- Can identify antigens specific to the patient's tumor.	- Complex and time-consuming method.
Immunopeptidomics	- Analyzing tumor cell lysates for peptides presented by MHC molecules to identify antigens recognized by the patient's immune system.	- Identifies antigens recognized by the patient's immune system.	- Complex and expensive method.

**Table 9:** Strategies for enhancing cancer immunotherapy: mechanisms, clinical outcomes, and challenges.

Interaction Type	Description	Mechanisms of Synergy	Clinical Outcomes	Challenges and Considerations
Combinatorial Treatment	Concurrent administration of cancer vaccines and checkpoint inhibitors.	- Vaccines prime the immune system, and checkpoint inhibitors release immune brakes. - Enhanced T-cell activation and tumor-specific response. - Reduced immunosuppression in the tumor microenvironment.	- Significant improvement in overall survival rates. - Markedly increased tumor regression and response rates. - Prolonged durability of response. - Lower risk of relapse.	- Risk of immune-related adverse events (irAEs) such as autoimmunity and inflammation. - Patient selection based on predictive biomarkers (e.g., PD-L1 expression). - Monitoring and management of irAEs.
Sequential Treatment	Administering cancer vaccines followed by checkpoint inhibitors or vice versa.	- Cancer vaccines sensitize tumors to checkpoint inhibitors. - Reinforce the immune response initiated by vaccines. - Amplify antigen presentation.	- Improved response rates when used sequentially. - Potential for durable responses and long-term remission. - Enhanced immune memory. - Enhanced tumor control in patients with minimal residual disease.	- Optimal timing and sequence require further investigation. - Balancing treatment-related toxicities and side effects. - Individualized treatment strategies based on patient characteristics.
Biomarker-Guided Strategies	Tailoring treatment based on patient-specific biomarkers (*e.g*., PD-L1 expression).	- Identifying patients likely to benefit from combination therapy. - Personalized treatment approaches. - Enhanced response rates in biomarker-positive patients.	- Marked improvement in response rates and survival in biomarker-positive patients. - Reduced adverse events and side effects in biomarker-negative patients. - More precise patient stratification for therapy.	- Limited availability of validated biomarkers. - Heterogeneity in biomarker expression within tumors. - The need for ongoing biomarker research and validation.
Combination with Other Therapies	Integrating cancer vaccines and checkpoint inhibitors with other treatments (e.g., chemotherapy, targeted therapy).	- Synergistic effects with multiple treatment modalities. - Potentiation of immune responses. - Enhanced antigen release and presentation.	- Improved overall survival and disease control. - Enhanced tumor shrinkage and immune infiltration. - Synergistic effects with targeted therapy in specific cancer subtypes.	- Potential for increased treatment-related toxicities and side effects. - Individualized treatment strategies based on patient characteristics and cancer type. - Monitoring for drug interactions and adverse events.
Checkpoint Blockade	Administering checkpoint inhibitors to block immune checkpoint proteins like PD-1 and CTLA-4.	- Blockade releases the brakes on immune responses. - Restores T-cell activity and tumor infiltration. - Potentiates vaccine-induced immune memory.	- Improved response rates and disease control. - Prolonged survival in various cancer types. - Enhanced immune memory and durable responses. - Enhanced vaccine-induced immune responses.	- Risk of immune-related adverse events (irAEs) such as autoimmunity and inflammation. - Patient selection based on predictive biomarkers (e.g., PD-L1 expression). - Monitoring and management of irAEs.
Cancer Vaccination	Administering cancer vaccines to stimulate an immune response against specific tumor antigens.	- Vaccines prime the immune system against tumor-specific antigens. - Enhance tumor antigen presentation. - Potentiate checkpoint inhibitor efficacy.	- Enhanced tumor-specific immune responses. - Potential for long-term control and prevention of relapse. - Synergy with checkpoint inhibitors in overcoming immunosuppression.	- Identifying relevant tumor antigens and personalization of vaccines. - Balancing immune activation with potential autoimmunity. - Vaccine delivery and formulation optimization.

**Table 10:** Emerging technologies for the development of cancer vaccines offer potential applications, advantages, and challenges.

Technology	Description	Potential Applications	Advantages	Challenges and Considerations
RNA Vaccines	Utilize messenger RNA (mRNA) to encode tumor antigens, triggering an immune response.	- Personalized cancer vaccines: Targeting patient-specific mutations. - Rapid vaccine development. - Diverse antigen targeting.	- Speedy vaccine production: Weeks, not months. - Customization based on individual mutations. - Minimal risk of genomic integration.	- mRNA stability and delivery challenges. - Cold storage requirements for certain formulations. - Long-term efficacy data still evolving.
Viral Vector Vaccines	Employ modified viruses as vectors to deliver tumor antigens and stimulate an immune response.	- Broad-spectrum cancer antigen targeting. - Durable immune memory. - Combination with checkpoint inhibitors.	- Robust and persistent immune responses. - Wide range of antigens for immune recognition. - Improved tumor antigen presentation.	- Potential immune responses against viral vectors. - Safety concerns regarding the use of viral vectors. - Pre-existing immunity in some patients.
Nanotechnology-Based Delivery Systems	Utilize nanoparticles and nanocarriers for targeted vaccine delivery, improving efficacy and precision.	- Precise antigen delivery to tumor sites. - Enhanced immune response. - Reduced off-target effects.	- Increased vaccine stability and antigen protection. - Controlled release of antigens. - Minimized toxicity and side effects.	- Nanoparticle clearance and biocompatibility issues. - Ensuring efficient antigen loading and controlled release. - Long-term safety concerns.
Artificial Intelligence (AI) in Design	Employ AI algorithms for vaccine design, predicting optimal antigen combinations for personalized vaccines.	- Personalized cancer vaccines. - Rapid identification of candidate antigens. - Accelerated development.	- Enhanced antigen selection accuracy. - Identification of rare or patient-specific antigens. - Accelerated vaccine design.	- Validation of AI predictions in clinical settings. - Ethical considerations regarding AI-driven personalized treatments. - Data privacy and security concerns.
CRISPR-Based Antigen Discovery	Utilize CRISPR technology to identify and modify cancer-specific antigens, enhancing their immunogenicity.	- Precise antigen editing and optimization. - Targeting previously unrecognized antigens.	- Customization of antigens for improved immune recognition. - Enhanced vaccine efficacy. - Targeting hidden or low-abundance antigens.	- Potential off-target effects and genomic stability concerns. - Ethical considerations regarding genome editing. - Long-term safety and regulatory scrutiny.

**Table 11:** presents the effective strategies for tackling obstacles in cancer vaccine development.

Challenge	Strategy	Specific Actions
Tumor Heterogeneity	Neoantigen Discovery	- Analyze genomic and transcriptomic data to identify patient-specific neoantigens. - Customize vaccines for each patient based on neoantigen profiles.
Immune Escape	Combination Therapies	- Combine cancer vaccines with immune checkpoint inhibitors to overcome immune evasion. - Enhance immune responses against tumor cells.
Antigen Selection	Predictive Algorithms	- Utilize AI-driven algorithms to predict and prioritize tumor antigens with high immunogenicity. - Refine antigen selection for vaccine development.
Vaccine Delivery	Nanocarrier Systems	- Employ nanotechnology for precise and targeted vaccine delivery to tumor sites. - Improve vaccine stability and protect antigens from degradation.
Immune Tolerance	Immunomodulatory Agents	- Administer immunomodulators to modulate immune responses and overcome immune tolerance mechanisms. - Enhance vaccine-induced immunity.
Clinical Trial Design	Biomarker-Guided Trials	- Design clinical trials based on predictive biomarkers, ensuring the enrolment of patients likely to benefit. - Stratify patients for personalized vaccines.
Regulatory Approvals	Collaboration and Data Sharing	- Collaborate with regulatory agencies, sharing comprehensive data on vaccine safety and efficacy. - Expedite approval processes for innovative vaccines.
Safety and Toxicity	Adverse Event Monitoring	- Implement rigorous monitoring for immune-related adverse events (irAEs). - Detect and manage potential toxicities early to ensure patient safety.
Vaccine Cost	Cost-Effective Manufacturing	- Develop cost-effective manufacturing processes for vaccines, optimizing production efficiency. - Reduce overall vaccine production costs.
Patient Access	Global Accessibility Strategies	- Develop strategies to ensure global access to innovative cancer vaccines, addressing healthcare disparities in vaccine availability. - Promote equity in healthcare delivery.

**Table 12:** A detailed examination of the ethical and regulatory aspects involved in the development of cancer vaccines.

Consideration	Description	Implementation and Considerations
Informed Consent	Ensuring that participants in clinical trials and vaccine studies provide informed and voluntary consent.	- Clear explanation of study objectives, procedures, and potential risks. - Ongoing communication with participants.
Ethical Recruitment	Adhering to ethical guidelines when recruiting participants, considering diverse populations and vulnerable individuals.	- Equitable recruitment strategies. - Protection of vulnerable groups. - Avoidance of undue influence or coercion.
Transparency	Maintaining transparency in research, including disclosure of conflicts of interest and funding sources.	- Publicly accessible study protocols and results. - Full disclosure of financial and non-financial interests.
Safety and Privacy of Participants	Protecting the safety and privacy of study participants and their health information throughout the research process.	- Stringent data protection measures. - Confidentiality safeguards for participant information. - Reporting of data breaches.
Regulatory Approvals and Compliance	Navigating regulatory processes and ensuring compliance with local and international regulations for vaccine development.	- Collaboration with regulatory authorities. - Timely and complete submission of documentation. - Adherence to ethical and legal requirements.
Data Sharing and Collaboration	Encouraging data sharing and collaboration among researchers to accelerate progress and validate findings.	- Establish data sharing agreements. - Promote open-access publication. - Collaborative research networks.
Access and Equity	Addressing issues of access and equity to ensure that promising vaccines are available to all, regardless of socio-economic status.	- Affordable pricing strategies. - Global distribution plans. - Equity in vaccine allocation during shortages.
Monitoring Adverse Events and Reporting	Establishing robust systems for monitoring and reporting adverse events during clinical trials and vaccine administration.	- Real-time monitoring of adverse events. - Immediate reporting to regulatory agencies. - Ongoing safety assessments.
Benefit-Risk Assessment and Communication	Conducting thorough benefit-risk assessments and effective communication of potential risks and benefits to participants.	- Transparent communication of risks and benefits. - Regular updates to participants and healthcare providers.
Long-term Follow-up and Post-Market Surveillance	Implementing long-term follow-up and post-market surveillance to assess vaccine safety and efficacy beyond initial trials.	- Extended monitoring for late-onset adverse events. - Surveillance for long-term efficacy and durability of response.
Regulatory Transparency and Timely Approvals	Promoting transparency in regulatory decision-making and expediting approvals for vaccines with significant benefits.	- Advocating for regulatory transparency. - Encouraging expedited review for vaccines with clear benefits. - Public engagement in regulatory processes.

## LIST OF ABBREVIATIONS

IARC: International Agency for Research on Cancer
PAP: Prostatic Acid Phosphatase
HPV: Human Papillomavirus
KEGG: Kyoto Encyclopedia of Genes and Genomes
HBV: Hepatitis B Virus
MHC: Major Histocompatibility Complex
WHO: World Health Organization
EMA: European Medicines Agency
FDA: Food and Drug Administration
GDEPT: Gene-Directed Enzyme Prodrug Therapy
TAA: Tumor-Associated Antigens
CTAs: Cancer Testis Antigens
TSAs: Tumor-Specific Antigens
OMVs: Outer Membrane Vesicles
HCC: Hepatocellular Carcinoma
GM-CSF: Granulocyte-Macrophage Colony-Stimulating Factor
T-VEC: Vaccine Talimogene Laherparepvec
ADEPT: Antibody-Directed Enzyme Prodrug Therapy
